# Avian Wing Proportions and Flight Styles: First Step towards Predicting the Flight Modes of Mesozoic Birds

**DOI:** 10.1371/journal.pone.0028672

**Published:** 2011-12-07

**Authors:** Xia Wang, Alistair J. McGowan, Gareth J. Dyke

**Affiliations:** 1 School of Biology and Environmental Science, University College Dublin, Belfield Dublin, Ireland; 2 School of Geographical and Earth Sciences, University of Glasgow, Glasgow, United Kingdom; 3 School of Ocean and Earth Science, National Oceanography Centre, University of Southampton, Southampton, United Kingdom; Zoological Society of London, United Kingdom

## Abstract

We investigated the relationship between wing element proportions and flight mode in a dataset of living avian species to provide a framework for making basic estimates of the range of flight styles evolved by Mesozoic birds. Our results show that feather length (*f*_prim_) and total arm length (*ta*) (sum of the humerus, ulna and manus length) ratios differ significantly between four flight style groups defined and widely used for living birds and as a result are predictive for fossils. This was confirmed using multivariate ordination analyses, with four wing elements (humerus, ulna/radius, manus, primary feathers), that discriminate the four broad flight styles within living birds. Among the variables tested, manus length is closely correlated with wing size, yet is the poorest predictor for flight style, suggesting that the shape of the bones in the hand wing is most important in determining flight style. Wing bone thickness (shape) must vary with wing beat strength, with weaker forces requiring less bone. Finally, we show that by incorporating data from Mesozoic birds, multivariate ordination analyses can be used to predict the flight styles of fossils.

## Introduction

The timing and sequence of events that led to the origin and subsequent evolution of flapping flight in birds remains an important unanswered question in vertebrate evolutionary biology. Despite a substantial increase in the numbers of early birds discovered and described, including exceptionally well-preserved dinosaurs with feathers, the single largest impediment to interpreting the flight adaptations of a fossil is the common absence of a preserved wing outline [1], [2]. Unlike other vertebrate groups that evolved powered flight (bats and pterosaurs), the leading edge of the avian wing is comprised, to a large part, of feathers which are less likely to fossilize. Thus, it has proved extremely difficult to validate in fossil species the correlations reported between the external wing morphology of living birds and flight performance [3]–[5]. Relatively little is known about *how* the proportions of the avian wing evolved [6] despite recent discoveries of numerous non-avian theropod dinosaurs with bird-like feathers [7]–[9].

A ‘functional wing’ (total arm, *ta*) in non-avian dinosaurs and birds is similarly comprised of the forelimb bones (i.e. humerus (*hu*), ulna *(ul*)/radius(*ra*) and manus (*mn*)) and the primary feathers (*f*_prim_). So far, the phylogenetic distribution of the bony components of the avian wing has been described [1], [10]–[12] and the contribution of feather length to wing length in both living and fossil birds has been analyzed [13]–[14]. The evolution of wing proportions in theropod dinosaurs and Mesozoic birds has also been studied by simple analyses of specimens with feathered arms [6].

Research that relates wing morphology to different flight modes among extant birds has been limited; Rayner (1988) [15] was the first to attempt to correlate flight styles to morphometric measurements by grouping flight styles by ecological niche. Much later, Nudds *et al.* [1] examined the brachial index (*hu*:*ul*/*ra*) within a sample of living birds and found that it differs between three different kinematic groups differentiated by their wing-beat frequencies. Although not directly related to flight, Hinic-Forlog and Motani [16] presented the results of an extensive multivariate analysis of 32 skeletal measurements that allowed them to predict the style of underwater locomotion in Cretaceous ocean-going hesperornithiforms.

Most recently, Simons [17] has shown that the wing bones of pelecaniform birds have specific morphologies that reflect the demands associated with different flight specializations; among these bones, the carpometacarpus (a compound bone situated between the knuckle and wrist that arose from the fusion of the metacarpals and distal carpals) was found to be most variable *between* different groups that have distinct modes of flight [17]. However, this study considered only a single lineage of extant birds; we cannot be sure how frequent the morphological character variation it utilizes is within other avian clades. Although wing bones are apparently robust predictors for flight mode in extant taxa [1], [10], few attempts have been made to extrapolate these predictions back into the avian fossil record. Previous studies have been restricted to analyses of the bony parts of the wing, without considering the primary feather component of wing length.

Here, we analyze a large sample of wing component measurements (including primary feathers) from extant and Mesozoic fossil bird groups. Our aim is to assess whether the proportions of the living avian wing are robust predictors of flight style that can then be applied to fossil taxa. Our specific objectives are: (1) to investigate the relationship between wing element proportions and flight modes across a wide range of living species; and (2) provide basic estimates for the range of flight styles used by Mesozoic birds based on parameters derived from our analyses of extant taxa.

## Materials and Methods

### Ethics Statement

“Living birds” here refers to museum specimens of extant Neornithes (not live birds) from the Natural History collections of the National Museum, Ireland. Mesozoic bird measurements we use were collected from fossil specimens in Chinese museums, as follows: Institute of Vertebrate Paleontology and Paleoanthropology (Beijing) and Shandong Tianyu Museum of Nature (Pingyi). Permission was granted by these institutions for specimen access and measurements.

### Measurements and Data

Measurements of wing bones and primary feathers were taken for a sample of living and Mesozoic birds (Table S1). Our living bird sample comprises 184 species from 55 families (18 orders) [10]. Measurements of Mesozoic birds were taken directly from fossil specimens (see above) and from publications [18] (Table S1). We measured the lengths of the *hu*, *ul*, *mn* and *f*_prim_ using digital calipers (rounded to the nearest mm) for both living and fossil birds; all variables were log_10_ transformed before analysis and means are displayed ± their standard error (SE).

Four flight styles for living birds were used:‘continuous flapping’ (CF) (e.g. grebes, ducks and auks); ‘flapping and soaring’ (FS) (e.g. storks, pelicans and large raptors); ‘flapping and gliding’ (FG) (e.g. swifts, falcons and gulls); ‘passerine-type flight’ (PT). These styles were coded for our living bird sample, as defined (and analyzed) by Bruderer *et al.* [19]. As our objective is to determine whether similarity in wing bone measurements, or *f*_prim_/*ta* ratios, are reliable predictors of flight styles, ANOVAs were applied to explore whether there are significant differences among the four flight styles. As group variances are not statistically equal, a Kruskal-Wallis non-parametric ANOVA was also employed. ANCOVAs were then used to remove the effects of both body size and wing size [13], [14], [17], with log_10_ transformed body weights and the geometric mean length of each of the four wing elements established as proxies for body size and wing size, respectively. If a particular range of values for manus (*mn*) length or *f*_prim_/*ta* ratio is indeed a reliable means of discriminating among flight styles then we should expect systematic, significant differences between the length of *mn* and the *f*_prim_/*ta* ratio across the four bird flight mode groups. As the object of our analysis is to test whether the taxa we sample here can be separated *into* any of the four flight-style groupings and not the analysis of evolutionary trends, we did not use phylogenetically independent contrasts. In any case, investigation of phylogenetic effects would be fruitless as our bird sample encompasses 18 orders with at least three binned in each flight style group. There is also no clear consensus regarding the inter-relationships of neornithine orders at present.

Given the large number of comparisons, Hochberg multiple comparisons tests (HMCT) were used *post hoc* to evaluate the robustness of significant results. Finally, Principal Components Analyses (PCA) on the covariance and Discriminant Function Analyses (DFA) based on linear combinations of predicted variables were performed to explore whether wing bone lengths and primary feather lengths are a robust means of ordinating and classifying taxa into the flight style groups of Bruderer *et al*. [19]. To remove the effects of total wing size in our multivariate analyses, the log_10_ transformed geometric mean of the four wing element variables was subtracted from each log_10_ transformed variable [17]. All tests were conducted using SPSS v. 18.0.1.

## Results

### Living birds

#### *f*_prim_/ta ratio and manus length analysis

**Figure 1:**
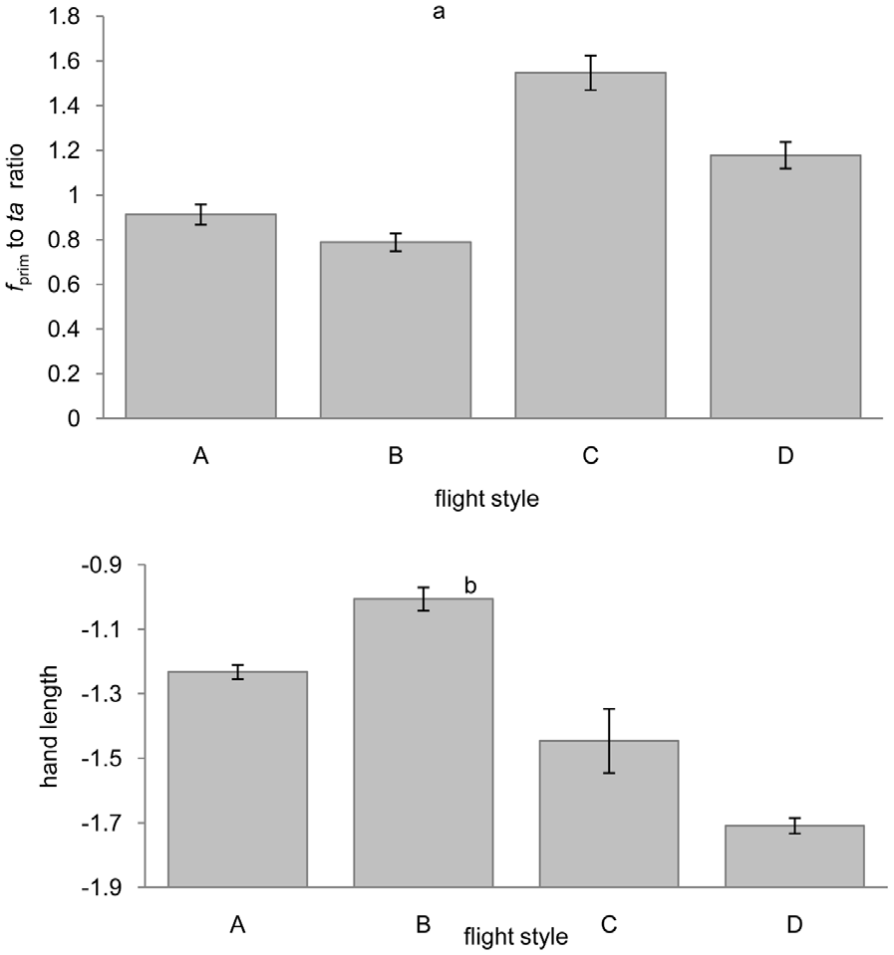
Mean primary feather length compared to total arm length (*f*_prim_/*ta*) and *mn* length for the four flight style groups of Bruderer et al. [18] (a) A one-way ANOVA showed that *f*_prim_/*ta* was significantly different between four flight styles (*F*_3,182_ = 37.789, *P*<0.001): back transformed mean *f*_prim_/*ta* ratio were group A = 0.9131 (0.8685, 0.9557), B = 0.7889 (0.7190, 0.8588), C = 1.5471 (1.0835, 2.0107) and D = 1.1782 (1.1319, 1.2244). (b) ANCOVA with body size controlled showed that *mn* length was significantly different between the four flight styles (*F*_3,182_ = 95.708, *P*<0.001): back transformed mean *mn* lengths for group A = −1.2326 (−1.2762, −1.1890), B = −1.0062 (−1.0823, −0.9301), C = −1.446 (−1.6814, −1.2114), D = −1.7088 (−1.7572, −1.6604).

One-way ANOVA and Kruskal-Wallis non-parametric tests show that *f*_prim_/*ta* ratios do differ significantly between the four flight style groups (*F*_3,182_ = 37.789, *P*<0.001; figure 1a). This does *not* reflect a body size effect because body mass (*M*) does not differ significantly between the four groups (*F*_3, 182_ = 0.136, *P = *0.939). Additional ANCOVAs with body size controlled and ANOVAs performed on the residuals (with body mass as independent proxy) corroborate this result (*F*_3, 182_ = 39.929, *P*<0.001; *F*_3, 182_ = 39.998, *P*<0.001). Although the error bars for groups ‘CF’ and ‘FS’ do not overlap (figure 1a), differences in these values are not significant enough to pass the HMCT (*P* = 0.062); thus, differences detected by ANOVAs must be attributable to differences between the other two groups. Specimens in groups ‘CF’ and ‘FS’ were then merged and a second HMCT comparing (‘CF’+‘FS’), ‘FG’ and ‘PT’ was performed: this test found a significant difference between the three groups (*F*_2, 180_ = 51.735, *P*<0.001).

Although our focus here is on the *f*_prim_/*ta* ratio, we also considered whether the length of other wing elements (i.e. *hu*, *ul*/*ra*, *mn* and *f*_prim_) was significantly different between the flight style groups. When body size was controlled for analysis in ANCOVA, all four wing elements were significantly different between the flight style groups (*hu*, *F*_3, 182_ = 113.139, *P*<0.001; *ul*, *F*_3, 182_ = 97.814, *P*<0.001; *mn*, *F*_3, 182_ = 95.305, *P*<0.001; *f_prim_ F*_3, 182_ = 80.215, *P*<0.001) with only *mn* length significantly different between any two of flight style groups after HMCT (*F*_3, 182_ = 95.708, *P*<0.001) (figure 1b). Also, an ANCOVA controlling for wing size (i.e. geometric mean from four wing element lengths) shows that *mn* length is not significantly different between the different flight style groups (*F*_3, 182_ = 1.773, *P* = 0.154). So, we conclude that *mn* length is closely correlated with wing size, and this parameter does vary significantly (*F*_3, 182_ = 99.955, *P*<0.001) among the four flight style groups.

### Principal component (PCA) and discriminant function (DFA) analysis

**Figure 2:**
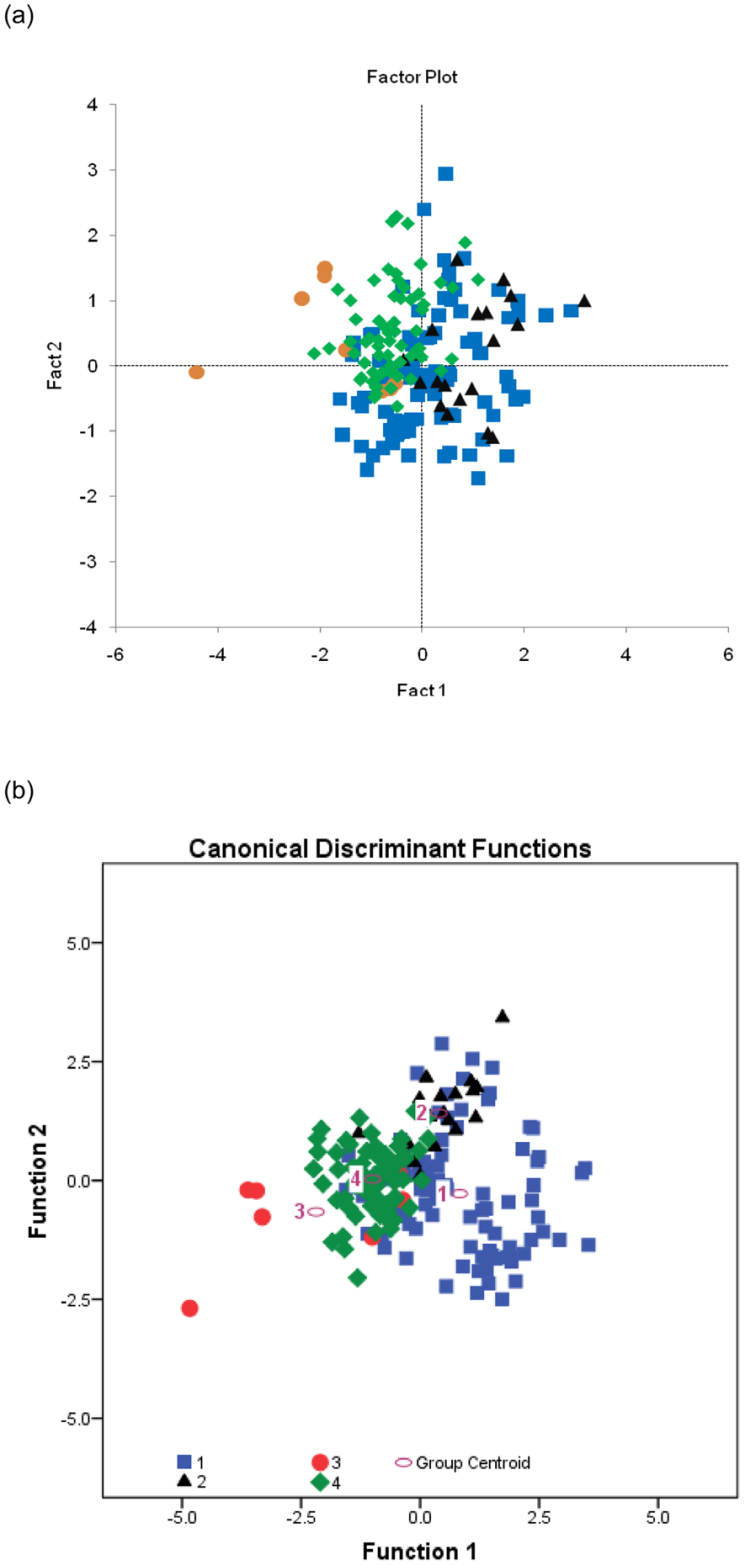
Multivariate analyses of four wing elements for 183 species (S1). (a) Principal component analysis (PCA) showed by (PC) 1 and 2. *hu* (0.897) and *ul* (0.822) have high and positive loadings on PC1,which exlplains 56% variance; *f*_prim_ has a high negative loading (−0.790) on PC1, which explains 32% variance; On PC 2, *mn* length has a high negative loading (−0.905); (b) Discriminant function analysis (DFA) represented by DFA functions 1 and 2. Function 1 is most strongly correlated with *hu* (−0.887) and *f*_prim_ (0.754) length and explains 76.3% variance; Function 2 explains 22.9% variance and is most strongly correlated with *ul* length (0.913). 1, ‘continuous flapping’; 2, ‘flapping and soaring’; 3, ‘flapping and gliding’; 4, ‘passerine-type flight’.

Both PCA and DFA (with four wing measurements included) can broadly discriminate between the four flight styles, but neither technique perfectly replicates the groupings (figure 2). Given that the categories themselves may not be sharply distinct, and some species may use different styles under different circumstances, some overlap in these categories is not unexpected. PCA shows that *hu* and *ul* have high and positive loadings on PC1 (0.897 and 0.822, respectively) while primary feather length has a high negative loading on PC1 (−0.790). On PC 2, *mn* length has a high negative loading (−0.905), while primary feather length has a high positive loading (0.612) (Table S2). PC1 can thus be used to approximately separate flight styles while no distinction can be discerned on PC2 (figure 2a). DFA differentiates flight styles much more clearly than does PCA (figure 2b), correctly classifying 72% of our sample (Table S3). Three canonical discriminant functions were used in the analysis: Function 1 is most strongly correlated with *hu* (−0.887) and *f*_prim_ (0.754) length and explains 76.3% of the variance; Function 2 explains 22.9% of the variance and is most strongly correlated with *ul* length (0.913); and Function 3, which is strongly correlated with *mn* length (−0.963), explains only 0.7% of the variance. In a stepwise DFA, *mn* length was the first variable to be removed because of it low explanatory power for grouping specimens (Table S3).

### Fossil birds

#### Results for *f*_prim_/ta, PCA and DFA plots

**Figure 3:**
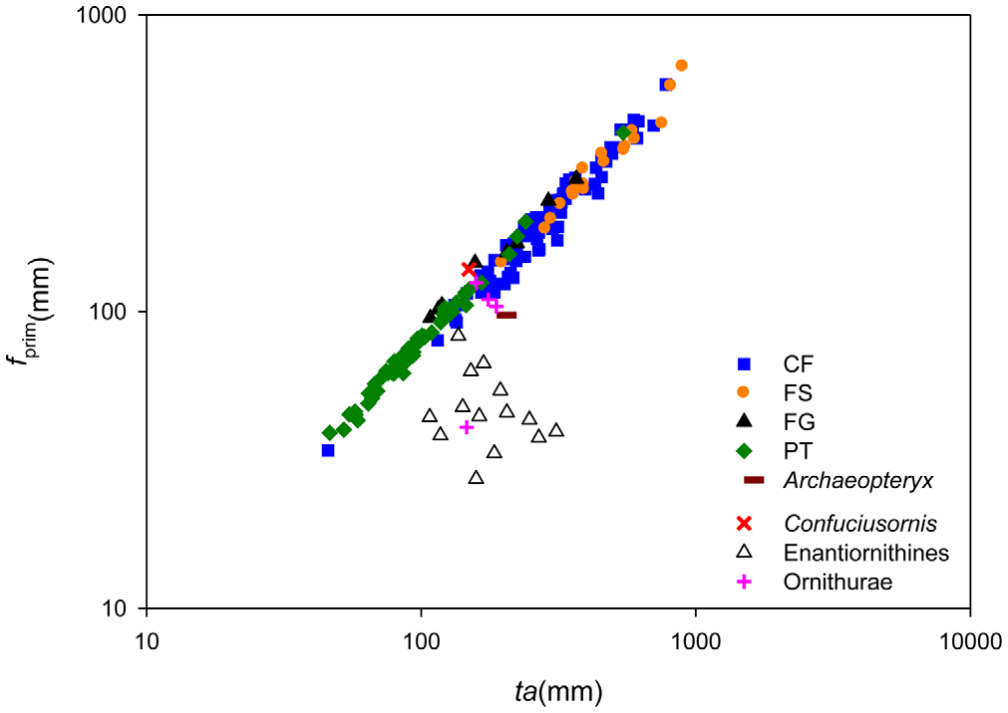
Predicted flight styles of fossil birds based on plots of primary feathers to total arm ratio (*f*_prim_/*ta*). CF, ‘continuous flapping’; FS, ‘flapping and soaring’; FG, ‘flapping and gliding’; PT, ‘passerine-type flight’; Ar (*Archaeopteryx*); Co (Confuciusornithidae); En (enantiornithines) and Or (Ornithurines). These results are summarized in Table 2.

**Figure 4:**
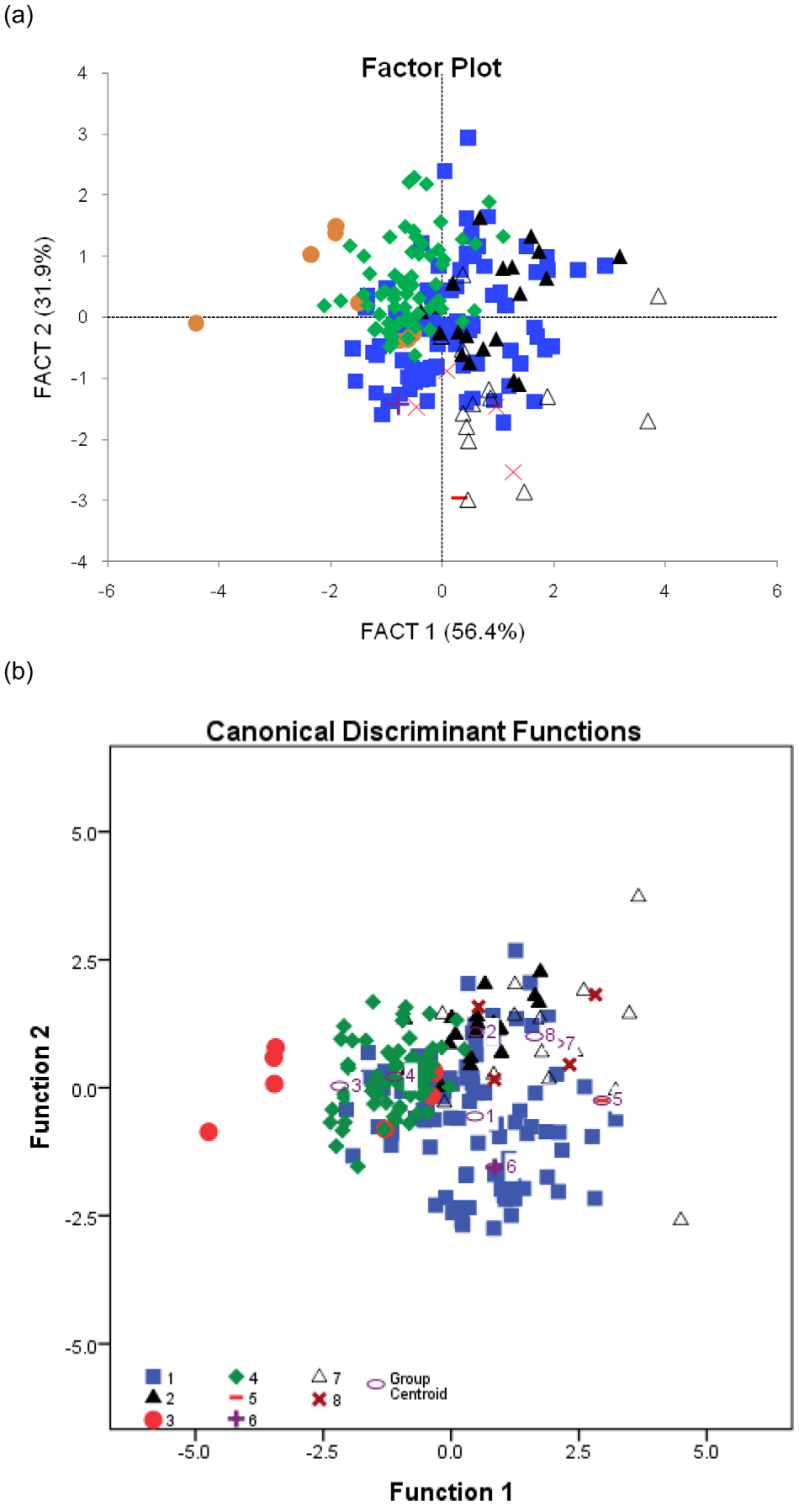
Predicted flight styles of fossil birds based on (a) Principal component analysis (PCA) and (b) Discriminant function analysis (DFA). 1,‘continuous flapping’; 2, ‘flapping and soaring’; 3, ‘flapping and gliding’; 4, ‘passerine-type flight’; 5, *Archaeopteryx*; 6, Confuciusornithidae; 7, enantiornithines and 8, Ornithurines. These results are summarized in Table 2.

As our analyses show that *mn* length is closely correlated with wing size, we attempted to infer the flight styles of fossil birds based on the plots of *f*_prim_/*ta*, PCA and DFA (Tables 1, 2; figures 3, 4).

**Table 1:** Posterior probabilities of discriminal functional analysis (DFA) for fossil birds.

	‘CF’	‘FS’	‘FG’	‘PT’
*Archaeopteryx*	0	0	0	0
*Confuciusornis*	0	0	0	0
Enantiornithines	21.4%	7.1%	0	0
Ornithurae	25%	0	0	0

CF, ‘continuous flapping’; FS, ‘flapping and soaring’; FG, ‘flapping and gliding’; PT, ‘passerine-type flight’.

**Table 2:** Predictions of flight styles for fossil birds in different plots.

	*f_prim_*/*ta*	PCA	DFA
*Archaeopteryx*	/	/	/
*Confuciusornis*	‘FG’	‘CF’	/
Enantiornithines	‘FG’	‘CF’, ‘FS’, ‘PT’	‘CF’, ‘FS’
Ornithurae	‘CF’, ‘PT’	‘CF’	‘CF’

*f*_prim_, average primary feather length; *ta*, average total arm length (humerus+ulna+hand); PCA, principal component analysis; DFA, discriminant function analysis; CF, ‘continuous flapping’; FS, ‘flapping and soaring’; FG, ‘flapping and gliding’; PT, ‘passerine-type flight’.

In summary, our results suggest that: (1) *Archaeopteryx* flew in a way quite different from modern birds (*f*_prim_/*ta* PCA and DFA) (figures 3, 4; Table S4); (2) *Confuciusornis* was either a member of the ‘CF’ group (PCA) or was a ‘flapping and gliding ’ (FG) bird (*f*_prim_/*ta*) (figures 3, Table S4); (3) sampled enantiornithines fall across the range of all defined flight styles; and (4) sampled members of Ornithurae overlap with the ‘CF’ and ‘PT’ groups.

Comparisons of *f*_prim_/*ta* ratios suggests that *Longipteryx chaoyangensis* overlaps with extant ‘CF’ birds andis close to the birds that use ‘FG’ flight(figure 3). PCA groups *Cathayornis sp.*, *Vescornis hebeiensis*, *Longirostravis hani* and *Hongshanornis longicresta* with extant ‘CF’, while *Longipteryx chaoyangensis* and *Alethoalaornis agitornis* overlap with extant ‘FG’ birds (figure 4). Posterior probabilities of DFA predicts *Eoenantiornis buhleri Concornis lacustrus*, *Vescornis hebeiensis* and *Longirostravis hani* as ‘CF’; predicts *Longipteryx chaoyangensis* as ‘FS’; and predicts *Archaeorhynchus spathula* as ‘CF’ (Table 1, Table S4; figure 4). (these results are summarized in Table 2).

## Discussion

### Wing elements and flight styles

A range of statistical and ordination techniques show that lengths of wing elements (*hu*, *ul*/ra, *mn*, *f*_prim_) are good predictors of flight behaviour among extant birds. This supports the obvious contention that wing bone morphology (e.g. length and mid-shaft diameters) reflects the demands of different types of aerial locomotion [17]. ANOVAs found no difference between groups ‘FG’ and ‘PT’ for *ta* (*P* = 0.079): the same result was found for both *hu* (*P* = 0.356) and *ul (P* = 0.384) (between groups ‘FG’ and ’PT’), while both *mn* (*P*<0.01) and primary feather lengths (*P*<0.001) do vary significantly. This result means that the similarities between flight style groups ‘FG’ and ‘PT’ are the result of forelimb length: wing size in these groups is determined by changes in forelimb length but difference in flight styles is controlled by primary feather and *mn* length. This is evidence that the ‘flapping and gliding’ group and the ‘passerine-type’ groups have similar forelimb lengths, but differ in the lengths of their primary feathers and *mn*. PCA results support that ‘continuous flapping’ birds and ‘flapping and gliding' birds differ in *ta* length but not in primary feather length, consistent with the earlier result that *f*_prim_ scales with negative allometry against *ta* [1], [14]: this simple relationship explains why primary feather length tends to be relatively shorter in birds with longer wings (Table S2).

### Manus length flexibility

After controlling for body size, *mn* length varies significantly between different flight style groups (figure 1b). However, further ANCOVAs controlling for wing size (*ta* length) show that *mn* length is not in fact significantly different among the different flight style groups. Nudds *et al.* [1] found *mn* length to be almost identical in different kinematic groups and so proposed that the relative length of this part of the hand-wing might not be correlated with distinct patterns of wing-movement. Our findings corroborate this argument: As wing lengths are significantly different (*P*<0.001) between the four flight style groups, and it is clear that *mn* length is strongly correlated with wing size, these results are consistent with studies that have found wing length related to flight style [3], [20]. A stepwise DFA found that *mn* length was the first variable to drop out while in the PCA, *mn* length was heavily loaded on PC2 (−0.905) but had low loadings on the other axes (Table S2). We interpret these results as evidence that *mn* length is varying freely in relation to other wing elements. This apparent flexibility is why it is the poorest predictor of flight style and not suitable for discriminating among the different types of flight.

Considering that the edge of the manus is the main attachment site for the primary feathers and the region of the airfoil surface that mediates drag and lift during flapping, we agree with Nudds *et al.* [1] that variation in *mn* length influences wing shape. However, it is other shape elements of the hand wing bones and not their overall length that is the most important of our variables for determining flight style.

### Relationship between *f*_prim_/*ta* ratio and flight styles

Our data show quantitatively that *f*_prim_/*ta* differs among groups of birds with different flight styles (figure 1a). It is noteworthy that such a simple ratio differs significantly among groups of birds with different flight styles, particularly when these groups are composed of birds from more than three different orders, with no significant differences in body mass (*F*_3, 182_ = 0.17, *P = *0.917. We suggest that, within the wing, variations in underlying bone ratios, rather than absolute sizes, permit a range of different flight styles by facilitating variation in upstroke kinematics [1]. Our results, based on a large sample of living species, show that that not only do bone length ratios vary but that the *f*_prim_/*ta* ratio can be correlated with broad flight styles. Of these, the ‘FG’ group has the highest *f*_prim_/*ta* ratio, followed by the ‘PT’ and the ‘CF’ group, while the ‘FS’ group has the lowest ratio (figure 1),which follow the general rule that larger living birds that soar (e.g. eagles, vultures, pelicans, and storks), tend to have longer wings with relatively shorter primaries, aiding in take off, while smaller birds (e.g. passerines and hawks) favour short wings and have relatively longer primary feathers, allowing for tight maneuvering in confined spaces [13], [20]. The result that enantiornithines lie outside *f*_prim_/*ta* ratio plots, with only *Longipteryx* overlapping with extant birds reflects the generally relatively shorter primary feathers in this group.

### Inferring the flight styles of fossil birds

Analysis of PCA, DFA and *f*_prim_/*ta* data produced three sets of interpretations for the flight styles of a range of Mesozoic taxa (Table 2). We are aware that while the ANOVA and ANCOVA work on the *f*_prim_/*ta* represents statistical testing, the PCA and DFA are ordination techniques that will always produce a result. With PCA this is the discovery of new axes that explain the variation, while DFA attempts to classify based on a weighted-sum of variables.

Nevertheless, both PCA and DFA results show that the flight style of *Archaeopteryx* was not comparable to living birds, which may simply be because it was an early-diverging flying bird with an unusual combination of traits, so it could not flap very well [21], disagreement with the view that *Archaeopteryx* was a powered flier [22], [23]. Results suggest that the flight style of *Confuciusornis* was comparable to ‘CF’ or ‘FG’ type, in contrast with earlier studies that suggest *Confuciusornis* could not flap well [21], [24], [25] and support the prediction that the very elongate wings, narrow primary rachises [21] and anatomy suggest that Confuciusornis was likely a glider [6].

The finding that enantiornithines plot across all the flight styles of living birds is in accordance with surveys that have shown the forelimb proportions of these birds to also fall within the range of extant taxa [26]. Within this clade *Vescornis*was classified by all analyses (i.e. PCA and DFA) as ‘CF’, while *Eoalulavis* and *Sinornis* were not classifiable in any of our three analyses. In recent phylogenetic studies [27], *Eoalulavis* and *Sinornis* were resolved as close to each other in the same polytomy, and thus may have possessed similar flight styles to one another, distinct from other enantiornithines.

Ornithurine birds are anatomically critical to understanding the later stages in the evolution of flapping flight because representatives of this lineage show development of a fused hand wing (carpometacarpus) for the first time [28]. However, Mesozoic ornithurines have remained rare [28]: to date, only five genera are known with feathers preserved. Of these, *Archaeorhynchus*, one of the most basal ornithurines, possessed a well-developed carpal trochlea on its carpometacarpus, a large wing and an alula [29] was classified as ‘CF’ by all three analyses. *Yixianornis* providing the earliest evidence of a tail fan similar to that seen in extant birds [30], and *Hongshanornis*, with long primaries and alula, being able to fly fast in open areas with a slim and unslotted wing [31] are classified as ‘CF’ in PCA (figure 4a). *Jianchangornis*, preserving an advanced pectoral girdle, sternum and wings and was thought likely capable of powerful flight [32], turn out to be overlapped with ‘CF’ birds in DFA (figure 4b).

In sum, our analysis show that the *f*_prim_/*ta* ratio is a useful metric for assessing flight styles in modern birds: four length variables (*hu*, *ul*/*ra*, *mn*, *f*_prim_) correctly classified 72% of our sample into one, or other, of the four flight style groups (Table S3). As a result we suggest that *f*_prim_/*ta* ratio and wing element lengths can be used as predictors for inferring the flight styles of fossil birds. Our results are one important first step towards reconstructing the functional ecomorphology, morphospace occupation and the roles that birds played in Meosozoic ecosystems.

## Supporting Information

Table S1 **Measurements for living birds and fossil birds used in analyses.**
(DOC)

Table S2 **Results of Principal Component Analysis (PCA).**
(DOC)

Table S3 **Results of Discriminant Function Analysis (DFA).**
(DOC)

Table S4 **Casewise statistics of fossil birds in Discriminant Function Analysis (DFA).**
(DOC)
