# Correction: Avian Wing Proportions and Flight Styles: First Step towards Predicting the Flight Modes of Mesozoic Birds

**DOI:** 10.1371/annotation/6c8c755f-58fc-49e3-b499-8d082318eef6

**Published:** 2013-10-24

**Authors:** Xia Wang, Alistair J. McGowan, Gareth J. Dyke

There were errors in Table S1. The correct version of the file is available here: 
