# Supplementary material for: Avian Wing Proportions and Flight Styles: First Step towards Predicting the Flight Modes of Mesozoic Birds
Source: PLoS One. 2011 Dec 7;6(12):e28672. doi: 10.1371/journal.pone.0028672 (PMC3233598; doi:10.1371/journal.pone.0028672)
Supplement: Table S1 — Measurements for living birds and fossil birds used in analyses. (DOC) [file pone.0028672.s001.doc]

**Wing proportions and flight style in birds: inferring the flight mode of Mesozoic fossil birds**

Xia Wang^1^, Alistair McGowan^2^ and Gareth J. Dyke^1,*^

**SUPPORTING MATERIAL**

**Table S1 Measurements for living birds and fossil birds used in analyses.**

| latin name | flight-type | *hu* (mm) | *ul* (mm) | *mn* (mm) | *f_prim_(*mm) | *ta*(mm) | *M* (g) |
| --- | --- | --- | --- | --- | --- | --- | --- |
| *Troglodytes aedon* | CF | 13.18 | 14.42 | 11.30 | 34 | 45.84 | 309 |
| *Coturnix coturnix* | CF | 35.76 | 29.94 | 23.63 | 80 | 114.77 | 955 |
| *Columba palumbus* | CF | 54.90 | 59.02 | 26.21 | 208 | 262.27 | 2138 |
| *Alle alle* | CF | 43.56 | 35.17 | 28.54 | 92 | 134.64 | 407 |
| *Upupa epops* | CF | 33.44 | 44.06 | 29.06 | 132 | 164.65 | 48 |
| *Podiceps nigricollis* | CF | 69.68 | 63.88 | 31.00 | 116 | 184.84 | 81 |
| *Charadrius hiaticula* | CF | 32.66 | 36.07 | 31.84 | 115 | 146.82 | 22 |
| *Rallus aquaticus* | CF | 40.24 | 32.12 | 32.37 | 94 | 133.34 | 447 |
| *Podiceps auritus* | CF | 77.65 | 67.93 | 33.85 | 124 | 200.85 | 309 |
| *Cepphus grylle* | CF | 60.18 | 50.46 | 34.20 | 150 | 209.45 | 219 |
| *Pica pica* | CF | 43.09 | 51.31 | 35.40 | 127 | 169.30 | 813 |
| *Calidris alpina* | CF | 28.07 | 29.36 | 36.39 | 105 | 132.22 | 6 |
| *Gallinula chloropus* | CF | 50.68 | 41.64 | 36.85 | 116 | 165.86 | 316 |
| *Streptopelia turtur* | CF | 36.92 | 41.14 | 37.78 | 149 | 185.19 | 1072 |
| *Lagopus mutus* | CF | 60.83 | 54.10 | 38.03 | 181 | 241.17 | 17 |
| *Limosa limosa* | CF | 62.46 | 67.19 | 38.21 | 180 | 241.80 | 15 |
| *Gallinago gallinago* | CF | 37.50 | 39.77 | 39.38 | 105 | 141.66 | 23 |
| *Lymnocryptes minimus* | CF | 31.75 | 33.98 | 40.79 | 105 | 135.91 | 23 |
| *Alectoris rufa* | CF | 53.43 | 49.80 | 41.15 | 121 | 173.64 | 324 |
| *Thalasseus sandvicensis* | CF | 69.00 | 82.50 | 41.20 | 278 | 346.50 | 2951 |
| *Athene noctua* | CF | 54.08 | 69.80 | 41.96 | 123 | 176.30 | 1023 |
| *Fratercula arctica* | CF | 63.26 | 50.77 | 42.78 | 114 | 176.46 | 427 |
| *Arenaria interpres* | CF | 39.91 | 43.12 | 44.74 | 136 | 175.17 | 537 |
| *Podiceps grisegena* | CF | 107.14 | 98.82 | 45.22 | 161 | 267.46 | 14 |
| *Aix galericulata* | CF | 70.10 | 58.00 | 45.60 | 191 | 260.47 | 115 |
| *Crex crex* | CF | 48.88 | 43.51 | 46.73 | 126 | 174.12 | 54 |
| *Columba livia* | CF | 44.62 | 51.34 | 47.00 | 193 | 237.00 | 18 |
| *Perdix perdix* | CF | 51.92 | 45.78 | 48.37 | 127 | 178.16 | 123 |
| *Columba oenas* | CF | 46.80 | 51.03 | 48.40 | 193 | 239.18 | 10 |
| *Alca torda* | CF | 76.91 | 59.00 | 48.87 | 131 | 207.16 | 110 |
| *Asio otus* | CF | 80.40 | 92.14 | 48.88 | 250 | 329.88 | 1000 |
| *Stercorarius parasiticus* | CF | 94.00 | 97.80 | 49.65 | 258 | 351.49 | 43 |
| *Corvus monedula* | CF | 47.36 | 60.46 | 49.92 | 204 | 250.77 | 2692 |
| *Anas crecca* | CF | 59.63 | 47.90 | 50.91 | 156 | 214.95 | 68 |
| *Anas clypeata* | CF | 75.04 | 64.53 | 51.63 | 208 | 282.45 | 457 |
| *Tyto alba* | CF | 82.52 | 91.41 | 51.75 | 230 | 311.97 | 13 |
| *Calidris canutus* | CF | 43.02 | 47.36 | 52.04 | 123 | 165.26 | 288 |
| *Tringa totanus* | CF | 44.60 | 49.41 | 52.07 | 125 | 168.85 | 3020 |
| *Caprimulgus europaeus* | CF | 37.74 | 48.51 | 53.31 | 167 | 204.09 | 302 |
| *Pluvialis apricaria* | CF | 48.66 | 54.41 | 54.56 | 169 | 217.01 | 2089 |
| *Pluvialis squatarola* | CF | 52.50 | 54.78 | 54.60 | 156 | 207.82 | 7413 |
| *Vanellus vanellus* | CF | 61.91 | 68.55 | 54.72 | 181 | 242.28 | 1660 |
| *Mergus serrator* | CF | 89.03 | 72.07 | 55.30 | 176 | 264.39 | 65 |
| *Bucephala clangula* | CF | 69.80 | 59.08 | 55.70 | 205 | 274.21 | 178 |
| *Strix aluco* | CF | 84.01 | 95.03 | 55.80 | 185 | 268.39 | 1023 |
| *Phalacrocorax aristotelis* | CF | 122.30 | 133.50 | 56.50 | 193 | 314.70 | 200 |
| *Aythya fuligula* | CF | 74.55 | 67.33 | 57.05 | 148 | 221.86 | 10715 |
| *Lagopus lagopus* | CF | 67.13 | 59.94 | 57.13 | 154 | 220.45 | 178 |
| *Otus scops* | CF | 67.07 | 86.41 | 57.75 | 124 | 190.32 | 219 |
| *Uria aalge* | CF | 87.25 | 63.13 | 58.89 | 130 | 216.53 | 891 |
| *Asio flammeus* | CF | 83.57 | 100.73 | 59.00 | 246 | 329.05 | 2042 |
| *Numenius arquata* | CF | 96.01 | 104.00 | 61.04 | 240 | 335.48 | 26 |
| *Pavo cristatus* | CF | 128.07 | 107.97 | 61.60 | 326 | 453.66 | 1072 |
| *Podiceps cristatus* | CF | 108.05 | 102.20 | 62.28 | 162 | 269.40 | 676 |
| *Fulica atra* | CF | 77.33 | 66.98 | 62.42 | 135 | 211.62 | 214 |
| *Anas platyrhynchos* | CF | 89.75 | 74.25 | 63.84 | 204 | 293.17 | 3311 |
| *Tetrao tetrix* | CF | 82.86 | 78.86 | 63.89 | 208 | 290.29 | 191 |
| *Rissa tridactyla* | CF | 85.60 | 94.60 | 64.48 | 238 | 323.08 | 14 |
| *Limosa lapponica* | CF | 60.59 | 64.53 | 65.17 | 163 | 222.95 | 17 |
| *Corvus frugilegus* | CF | 66.98 | 81.93 | 67.68 | 270 | 336.50 | 275 |
| *Somateria mollissima* | CF | 111.80 | 97.13 | 69.23 | 190 | 301.21 | 10 |
| *Corvus corone* | CF | 66.26 | 79.50 | 69.63 | 228 | 293.73 | 5495 |
| *Scolopax rusticola* | CF | 52.90 | 57.42 | 69.91 | 156 | 208.25 | 24 |
| *Nycticorax nycticorax* | CF | 107.31 | 119.48 | 70.37 | 216 | 322.76 | 11 |
| *Aythya ferina* | CF | 86.46 | 72.37 | 71.44 | 153 | 238.81 | 120 |
| *Anas penelope* | CF | 86.22 | 72.51 | 72.87 | 197 | 282.65 | 14 |
| *Haematopus ostralegus* | CF | 73.47 | 77.75 | 72.91 | 190 | 262.89 | 129 |
| *Larus ridibundus* | CF | 76.81 | 88.81 | 73.77 | 237 | 313.31 | 479 |
| *Burhinus oedicnemus* | CF | 80.26 | 90.34 | 74.88 | 230 | 309.75 | 11 |
| *Recurvirostra avosetta* | CF | 73.48 | 77.27 | 76.55 | 190 | 262.90 | 457 |
| *Ardea purpurea* | CF | 138.42 | 159.15 | 76.59 | 258 | 395.95 | 132 |
| *Nyctea scandiaca* | CF | 156.00 | 169.00 | 84.00 | 340 | 495.63 | 21 |
| *Larus canus* | CF | 83.05 | 91.64 | 84.48 | 282 | 364.62 | 1778 |
| *Tetrao urogallus* | CF | 118.24 | 113.12 | 85.95 | 263 | 380.78 | 631 |
| *Egretta alba* | CF | 152.40 | 177.53 | 91.23 | 320 | 472.01 | 282 |
| *Gavia stellata* | CF | 139.63 | 112.89 | 91.33 | 174 | 313.06 | 30 |
| *Anas acuta* | CF | 90.60 | 78.78 | 95.52 | 234 | 324.12 | 389 |
| *Branta canadensis* | CF | 180.77 | 161.33 | 99.50 | 438 | 618.50 | 759 |
| *Branta leucopsis* | CF | 132.53 | 120.55 | 99.72 | 358 | 490.19 | 107 |
| *Gavia immer* | CF | 191.68 | 153.05 | 99.74 | 250 | 441.22 | 20 |
| *Larus argentatus* | CF | 128.77 | 144.26 | 103.82 | 305 | 433.39 | 62 |
| *Phalacrocorax carbo* | CF | 161.11 | 174.07 | 105.57 | 269 | 429.69 | 295 |
| *Bubo bubo* | CF | 157.79 | 183.77 | 112.13 | 358 | 515.46 | 162 |
| *Larus marinus* | CF | 153.27 | 170.55 | 120.16 | 442 | 595.02 | 22 |
| *Ardea cinerea* | CF | 170.73 | 200.77 | 120.78 | 284 | 454.34 | 83 |
| *Branta bernicla* | CF | 123.57 | 113.08 | 122.47 | 410 | 533.29 | 54 |
| *Otis tarda* | CF | 196.46 | 215.42 | 177.16 | 582 | 778.34 | 28 |
| *Anser anser* | CF | 179.74 | 171.11 | 181.19 | 410 | 589.51 | 4786 |
| *Grus grus* | CF | 227.16 | 247.46 | 192.50 | 384 | 610.93 | 468 |
| *Cygnus olor* | CF | 279.68 | 254.68 | 231.93 | 423 | 702.50 | 93 |
| *Accipiter nisus* | FS | 51.77 | 62.29 | 49.74 | 146 | 197.06 | 8128 |
| *Bubulcus ibis* | FS | 94.00 | 106.88 | 50.15 | 190 | 283.38 | 18 |
| *Plegadis falcinellus* | FS | 93.30 | 101.30 | 54.50 | 205 | 297.71 | 40 |
| *Accipiter gentilis* | FS | 94.21 | 105.05 | 78.86 | 273 | 366.75 | 66 |
| *Circus cyaneus* | FS | 92.34 | 104.42 | 79.37 | 230 | 321.83 | 1549 |
| *Botaurus stellaris* | FS | 134.29 | 143.18 | 81.91 | 258 | 391.82 | 155 |
| *Milvus migrans* | FS | 117.24 | 133.85 | 90.43 | 340 | 456.88 | 457 |
| *Circus pygargus* | FS | 86.58 | 116.88 | 92.76 | 303 | 389.17 | 562 |
| *Buteo buteo* | FS | 106.88 | 127.15 | 94.35 | 253 | 359.42 | 145 |
| *Aquila chrysaetos* | FS | 184.56 | 214.78 | 95.47 | 405 | 589.26 | 339 |
| *Buteo lagopus* | FS | 112.92 | 131.31 | 97.73 | 247 | 359.46 | 4169 |
| *Circus aeruginosus* | FS | 104.11 | 126.34 | 100.43 | 253 | 356.65 | 776 |
| *Ciconia nigra* | FS | 196.00 | 217.00 | 107.33 | 359 | 554.67 | 1023 |
| *Milvus milvus* | FS | 123.58 | 141.42 | 112.82 | 268 | 391.16 | 3467 |
| *Pandion haliaetus* | FS | 146.40 | 184.99 | 124.70 | 319 | 465.05 | 16 |
| *Gypaetus barbatus* | FS | 225.81 | 266.36 | 135.88 | 670 | 895.71 | 79 |
| *Pelecanus onocrotalus* | FS | 325.00 | 371.00 | 139.20 | 430 | 754.76 | 11 |
| *Gyps fulvus* | FS | 237.85 | 292.02 | 161.55 | 576 | 813.71 | 13 |
| *Ciconia ciconia* | FS | 199.33 | 229.92 | 169.04 | 350 | 549.04 | 95 |
| *Haliaeetus albicilla* | FS | 220.24 | 248.99 | 186.20 | 381 | 601.00 | 20 |
| *Delichon urbica* | FG | 13.98 | 21.30 | 16.57 | 95 | 108.03 | 891 |
| *Riparia riparia* | FG | 14.50 | 21.12 | 17.89 | 102 | 115.58 | 14 |
| *Hirundo rustica* | FG | 14.98 | 23.41 | 20.56 | 105 | 119.08 | 550 |
| *Apus apus* | FG | 11.83 | 18.11 | 27.93 | 146 | 157.07 | 2089 |
| *Falco columbarius* | FG | 47.58 | 53.82 | 50.95 | 158 | 204.90 | 9 |
| *Falco tinnunculus* | FG | 53.85 | 63.41 | 55.37 | 170 | 223.21 | 15 |
| *Falco subbuteo* | FG | 55.32 | 63.03 | 60.50 | 236 | 290.79 | 1585 |
| *Falco peregrinus* | FG | 87.26 | 102.32 | 92.39 | 280 | 366.83 | 9550 |
| *Regulus regulus* | PT | 8.82 | 12.72 | 8.22 | 39 | 46.49 | 479 |
| *Certhia familiaris* | PT | 12.80 | 15.80 | 8.37 | 53 | 64.59 | 60 |
| *Parus ater* | PT | 12.70 | 15.10 | 8.70 | 46 | 57.44 | 575 |
| *Sylvia curruca* | PT | 13.10 | 15.80 | 8.80 | 60 | 71.94 | 34 |
| *Parus montanus* | PT | 13.60 | 16.30 | 9.00 | 40 | 52.29 | 240 |
| *Aegithalos caudatus* | PT | 11.00 | 13.85 | 11.00 | 45 | 54.75 | 50 |
| *Acrocephalus schoenobaenus* | PT | 12.69 | 15.17 | 12.38 | 63 | 74.57 | 912 |
| *Saxicola torquata* | PT | 15.75 | 19.88 | 12.84 | 53 | 67.57 | 288 |
| *Acanthis cannabina* | PT | 17.03 | 20.99 | 13.93 | 43 | 58.79 | 19 |
| *Parus caeruleus* | PT | 13.95 | 17.01 | 14.45 | 45 | 57.72 | 2951 |
| *Muscicapa striata* | PT | 15.40 | 21.70 | 14.55 | 70 | 84.33 | 22 |
| *Alcedo atthis* | PT | 25.16 | 29.45 | 14.74 | 62 | 86.04 | 36 |
| *Phoenicurus phoenicurus* | PT | 15.99 | 20.34 | 14.81 | 75 | 89.95 | 437 |
| *Parus major* | PT | 16.42 | 20.00 | 14.86 | 49 | 64.22 | 617 |
| *Acrocephalus scirpaceus* | PT | 11.93 | 16.84 | 14.97 | 57 | 67.79 | 112 |
| *Erithacus rubecula* | PT | 16.39 | 19.52 | 15.01 | 51 | 66.20 | 19 |
| *Motacilla cinerea* | PT | 18.07 | 23.77 | 15.08 | 78 | 95.04 | 692 |
| *Carduelis chloris* | PT | 19.03 | 23.37 | 15.28 | 74 | 91.98 | 1000 |
| *Bombycilla garrulus* | PT | 22.20 | 27.40 | 15.50 | 101 | 122.27 | 162 |
| *Jynx torquilla* | PT | 23.41 | 27.25 | 15.51 | 71 | 93.35 | 9 |
| *Prunella modularis* | PT | 16.44 | 17.99 | 15.55 | 54 | 69.28 | 891 |
| *Picoides minor* | PT | 21.20 | 24.09 | 15.65 | 68 | 88.12 | 28 |
| *Carduelis spinus* | PT | 12.98 | 15.94 | 15.71 | 68 | 79.90 | 1698 |
| *Passer montanus* | PT | 16.79 | 18.92 | 15.71 | 64 | 79.70 | 17 |
| *Saxicola rubetra* | PT | 16.18 | 22.06 | 16.05 | 67 | 82.09 | 4169 |
| *Motacilla alba* | PT | 19.21 | 23.90 | 16.28 | 74 | 92.16 | 871 |
| *Pyrrhula pyrrhula* | PT | 18.94 | 23.27 | 16.39 | 62 | 79.83 | 18 |
| *Anthus pratensis* | PT | 19.52 | 23.85 | 16.65 | 78 | 96.50 | 15 |
| *Luscinia svecica* | PT | 16.02 | 20.87 | 17.72 | 63 | 77.93 | 162 |
| *Sitta europaea* | PT | 18.53 | 22.32 | 18.08 | 68 | 85.47 | 933 |
| *Ficedula hypoleuca* | PT | 14.85 | 21.82 | 18.10 | 62 | 75.76 | 240 |
| *Emberiza schoeniclus* | PT | 17.96 | 20.59 | 18.77 | 62 | 78.86 | 68 |
| *Luscinia megarhynchos* | PT | 17.00 | 22.93 | 18.81 | 81 | 97.00 | 575 |
| *Plectrophenax nivalis* | PT | 20.20 | 23.65 | 18.85 | 103 | 122.29 | 776 |
| *Sylvia atricapilla* | PT | 16.97 | 20.01 | 19.00 | 63 | 78.88 | 8 |
| *Cinclus cinclus* | PT | 22.00 | 24.90 | 19.20 | 82 | 103.01 | 832 |
| *Emberiza cirlus* | PT | 17.70 | 19.65 | 19.37 | 67 | 83.64 | 339 |
| *Lanius collurio* | PT | 20.18 | 23.85 | 19.60 | 80 | 99.18 | 1445 |
| *Fringilla coelebs* | PT | 18.16 | 22.43 | 20.01 | 65 | 82.09 | 724 |
| *Emberiza citrinella* | PT | 20.52 | 24.04 | 20.25 | 68 | 87.47 | 14 |
| *Passer domesticus* | PT | 19.09 | 22.45 | 20.29 | 63 | 81.01 | 407 |
| *Lanius excubitor* | PT | 26.86 | 33.02 | 20.77 | 92 | 117.91 | 1096 |
| *Loxia curvirostra* | PT | 20.50 | 25.24 | 20.77 | 81 | 100.51 | 3020 |
| *Fringilla montifringilla* | PT | 18.87 | 22.86 | 20.83 | 83 | 100.89 | 29 |
| *Carduelis carduelis* | PT | 16.85 | 22.98 | 21.19 | 63 | 78.78 | 4169 |
| *Turdus iliacus* | PT | 26.51 | 31.88 | 21.25 | 93 | 118.57 | 12 |
| *Lanius senator* | PT | 21.65 | 29.66 | 21.65 | 73 | 93.63 | 263 |
| *Tichodroma muraria* | PT | 19.98 | 25.90 | 21.95 | 70 | 88.94 | 1072 |
| *Coccothraustes coccothraustes* | PT | 23.64 | 26.77 | 23.64 | 98 | 120.72 | 832 |
| *Alauda arvensis* | PT | 25.16 | 30.63 | 24.01 | 85 | 109.20 | 138 |
| *Turdus merula* | PT | 29.67 | 35.43 | 28.82 | 98 | 126.78 | 23 |
| *Picoides major* | PT | 32.02 | 36.79 | 29.58 | 118 | 149.19 | 56 |
| *Turdus philomelos* | PT | 26.83 | 30.95 | 29.72 | 92 | 117.91 | 115 |
| *Sturnus vulgaris* | PT | 27.77 | 33.73 | 30.97 | 107 | 133.91 | 490 |
| *Oriolus oriolus* | PT | 31.95 | 40.84 | 31.45 | 119 | 150.13 | 490 |
| *Turdus pilaris* | PT | 29.97 | 34.58 | 31.81 | 116 | 145.14 | 407 |
| *Monticola solitarius* | PT | 25.84 | 35.77 | 33.79 | 97 | 121.95 | 380 |
| *Turdus viscivorus* | PT | 31.24 | 35.02 | 35.02 | 114 | 144.41 | 355 |
| *Picus viridis* | PT | 41.55 | 48.08 | 35.03 | 105 | 145.70 | 347 |
| *Garrulus glandarius* | PT | 41.68 | 49.06 | 36.06 | 125 | 165.89 | 339 |
| *Turdus torquatus* | PT | 30.73 | 40.82 | 37.61 | 100 | 129.87 | 525 |
| *Cuculus canorus* | PT | 39.74 | 44.04 | 45.81 | 201 | 240.13 | 18 |
| *Dryocopus martius* | PT | 54.00 | 60.00 | 50.00 | 156 | 209.31 | 200 |
| *Coracias garrulus* | PT | 45.40 | 57.44 | 54.93 | 179 | 223.77 | 32 |
| *Neophron percnopterus* | PT | 143.45 | 161.65 | 75.95 | 401 | 544.13 | 5623 |

| latin name | Specimen No./Source | Humerus (mm) | forearm (mm) | hand (mm) | *fprim(mm)* | *ta(mm)* |
| --- | --- | --- | --- | --- | --- | --- |
| *Archaeopteryx* | [17] | 61.93 | 54.91 | 71.48 | 97.30 | 167.94 |
| *Confuciusornis* | pers.obs. | 49.32 | 44.84 | 55.09 | 138.79 | 117.30 |
| *Eoenantiornis buhleri* | IVPP V11537/pers.obs | 29.43 | 31.17 | 24.86 | 66.91 | 168.95 |
| *Alethoalaornis agitornis* | LPM 00009/[1] | 24.00 | 26.00 | 19.60 | 33.30 | 184.81 |
| *Concornis lacustrus* | LH2814/[2] | 68.10 | 38.90 | 15.00 | 47.70 | 141.71 |
| *Dapingfangornis sentisorhinus* | LPM 00039[3] | 22.00 | 27.00 | 22.20 | 44.40 | 162.91 |
| *Eoalulavis hoyasi* | LH 13500/[4] | 30.00 | 35.10 | 19.00 | 27.30 | 158.45 |
| *Longipteryx chaoyangensis* | STM A8-3/pers.obs. | 28.50 | 36.77 | 29.52 | 82.84 | 136.56 |
| *Longirostravis hani* | IVPP V11309/pers.obs. | 23.28 | 24.81 | 16.02 | 62.96 | 151.84 |
| *Protopteryx fengningensis* | IVPP V11665/pers.obs. | 26.89 | 26.76 | 27.40 | 44.09 | 107.50 |
| *Shanweiniao cooperorum* | DNHM D1878/1/[5] | 22.43 | 23.46 | 23.36 | 43.20 | 248.11 |
| *Vescornis hebeiensis* | NIGP 130722/[6] | 25.20 | 24.60 | 22.60 | 45.70 | 205.21 |
| *Paraprotopteryx gracilisi* | STM 1-7/pers.obs. | 18.39 | 21.45 | 20.00 | 39.38 | 310.63 |
| *Cathayornis sp.* | STM A11-58/pers.obs. | 21.12 | 23.25 | 18.87 | 37.58 | 268.19 |
| *Cuspirostrisornis houi* | STM A11-65/pers.obs. | 26.49 | 23.36 | 28.70 | 38.25 | 117.56 |
| *Sinornis santensis* | STM A11-66/pers.obs. | 44.84 | 44.06 | 42.37 | 54.21 | 194.36 |
| *Hongshanornis longicresta* | IVPP V14533/pers.obs. | 25.96 | 25.08 | 20.80 | 40.74 | 146.62 |
| *Yixianornis grabaui* | IVPP V12631/pers.obs. | 38.84 | 49.06 | 52.06 | 104.05 | 187.94 |
| *Jianchangornis microdonta* | IVPP V16708/pers.obs. | 76.20 | 82.10 | 80.30 | 109.80 | 175.56 |
| *Archaeorhynchus spathula* | IVPP V14287/pers.obs. | 53.00 | 56.00 | 50.00 | 125.00 | 158.94 |

Abbreviations: *hu*, humerus; *ul*, ulna; *mn*, manus; *f*_prim_, average primary feather length; *ta*, average total arm length (humerus+ulna+hand); *M*, body mass.Four flight styles -CF, ‘continuous flapping’; FS, ‘flapping and soaring’; FG, ‘flapping and gliding’; PT, ‘passerine-type flight’; Institutional acronyms (China): DNHM, Dalian Natural History Museum (Dalian); GMV, NGMC, National Geological Museum of China (Beijing); IVPP, Institute of Vertebrate Paleontology and Paleoanthropology (Beijing); LH, refers to the collection from Las Hoyas; LPM, Liaoning Provincial Museum (Beipiao); NIGP, Nanjing Institute of Geology and Paleontology, Chinese Academy of Science (Nanjing); STM, Shandong Tianyu Museum of Nature (Pingyi).

**Additional references**

1. Li, L., Hu, D.-Y., Duan, Y., Gong, E.-P.& Hou, L.-H. 2007 Alethoalaornithidae Fam. Nov: a new family of enantiornithine bird from the lower Cretaceous of western Liaoning. *Acta Palaeontologica Sinica* **46**, 365-372.
2. Sanz, J, L,, Chiappe, L. M. & Buscalioni, A. 1995 The osteology of *Concornis lacustris* (Aves: Enantiornithes) from the Lower Cretaceous of Spain and a re-examination of its phylogenetic relationships. *Am. Mus. Novit.* **3133**, 1–23.
3. Li, L., Duan, Y., Hu, D.-Y., Wang, L., Cheng, S.-L. & Hou, L.-H. 2006 New Eoenantiornithid bird from the Early Cretaceous Jiufotang Formation of western Liaoning, China. *Acta Geologica Sinica* (English edition) **80**, 38–41.
4. Sanz, J. L., Chiappe, L. M., Pérez-Moreno B. P., Buscalioni, A. D. & Moratalla, J. 1996 A Lower Cretaceous bird from Spain: implications for the evolution of flight. *Nature* **382**, 442–445.
5. O'Connor, J. K., Wang, X.-R., Chiappe, L. M., Gao, C.-H., Meng, Q.-J., Cheng, X.-D., & Liu, J.-Y. 2009 Phylogenetic support for a specialized clade of Cretaceous enantiornithine birds with information from a new species. *Journal of Vertebrate Paleontology* **29**, 188–204.
6. Zhang, F.-C., Ericson, P.G.P. & Zhou, Z.-H. 2004 Description of a new enantiornithine bird from the Early Cretaceous of Hebei, northern China. *Canadian Journal of Earth Sciences* **41**, 1097-1107.
