# Supplementary material for: Avian Wing Proportions and Flight Styles: First Step towards Predicting the Flight Modes of Mesozoic Birds
Source: PLoS One. 2011 Dec 7;6(12):e28672. doi: 10.1371/journal.pone.0028672 (PMC3233598; doi:10.1371/journal.pone.0028672)
Supplement: Table S2 — Results of Principal Component Analysis (PCA). (DOC) [file pone.0028672.s002.doc]

**Table S2. Results of Principal Component Analysis**

| **Explained Variance (Eigenvalues)** | | | |  |  | **Unrotated Factor Loadings** | | |
| --- | --- | --- | --- | --- | --- | --- | --- | --- |
| Value | Factor 1 | Factor 2 | Factor 3 | Factor 4 |  | Variable | Factor 1 | Factor 2 |
| Eigenvalue | 2.257 | 1.278 | 0.466 | 0.000 |  | humerus | 0.897 | -0.105 |
| % of Var. | 56.422 | 31.939 | 11.639 | 0.000 |  | ulna | 0.822 | 0.271 |
| Cum. % | 56.422 | 88.361 | 100.000 | 100.000 |  | hand | -0.391 | -0.905 |
|  |  |  |  |  |  | *fprim* | -0.790 | 0.612 |
| **Casewise Factor Scores of Principal Component Analysis** | | |  |  |  |  |  |  |
| Case | Factor 1 | Factor 2 | Case | Factor 1 | Factor 2 | Case | Factor 1 | Factor 2 |
| 1 | 0.169 | -0.221 | 70 | -0.368 | -1.018 | 139 | -0.603 | -0.039 |
| 2 | 0.264 | -0.072 | 71 | 1.788 | 0.743 | 140 | 0.028 | 0.941 |
| 3 | 0.462 | 2.941 | 72 | 1.501 | 1.160 | 141 | -1.816 | 0.269 |
| 4 | 0.309 | -0.288 | 73 | -0.788 | -0.180 | 142 | -0.852 | 0.398 |
| 5 | -0.382 | 1.211 | 74 | 0.488 | -0.211 | 143 | -0.549 | 0.667 |
| 6 | 1.875 | 0.994 | 75 | 1.695 | 0.738 | 144 | -0.417 | 1.033 |
| 7 | -0.827 | 0.077 | 76 | 1.657 | -1.381 | 145 | -0.114 | 0.537 |
| 8 | -0.393 | -0.852 | 77 | -0.962 | -1.372 | 146 | -0.544 | 1.069 |
| 9 | 1.885 | 0.774 | 78 | 0.571 | 1.009 | 147 | -0.846 | 0.163 |
| 10 | 0.325 | 0.773 | 79 | -0.243 | 0.043 | 148 | -0.640 | 0.382 |
| 11 | 0.147 | 0.439 | 80 | 1.972 | -0.475 | 149 | -0.670 | 0.162 |
| 12 | -1.553 | -1.050 | 81 | 0.528 | -0.109 | 150 | -0.781 | -0.100 |
| 13 | -0.084 | -0.445 | 82 | 1.698 | -0.315 | 151 | -1.299 | 0.714 |
| 14 | -1.340 | 0.254 | 83 | 0.900 | 0.362 | 152 | -1.651 | 1.169 |
| 15 | -0.066 | 0.844 | 84 | 0.095 | 0.431 | 153 | -0.970 | -0.243 |
| 16 | 0.524 | 1.320 | 85 | 1.840 | -0.525 | 154 | -0.845 | 0.688 |
| 17 | -0.541 | -0.831 | 86 | -1.241 | -0.567 | 155 | -1.210 | -0.212 |
| 18 | -1.184 | -1.238 | 87 | -0.245 | -0.076 | 156 | -1.029 | 0.426 |
| 19 | 0.243 | -0.434 | 88 | -0.269 | -1.372 | 157 | -0.842 | -0.141 |
| 20 | 0.043 | 2.396 | 89 | 1.194 | -1.124 | 158 | -0.618 | 0.006 |
| 21 | 1.118 | 0.195 | 90 | 1.098 | -1.721 | 159 | -0.614 | -0.341 |
| 22 | 0.557 | -0.739 | 91 | -0.045 | -0.247 | 160 | -0.067 | 1.102 |
| 23 | -1.179 | -0.615 | 92 | 1.736 | 1.086 | 161 | -0.861 | 0.304 |
| 24 | 2.427 | 0.778 | 93 | 1.256 | 0.827 | 162 | -1.329 | 0.192 |
| 25 | -0.256 | 0.452 | 94 | -0.043 | 0.188 | 163 | -0.910 | -0.309 |
| 26 | -0.622 | -0.978 | 95 | 0.294 | -0.231 | 164 | -0.221 | 1.016 |
| 27 | -1.341 | 0.362 | 96 | 1.395 | 0.397 | 165 | -0.106 | 0.248 |
| 28 | -0.482 | -1.026 | 97 | 0.193 | 0.566 | 166 | -0.625 | -0.087 |
| 29 | -1.369 | 0.158 | 98 | -0.370 | 0.105 | 167 | -1.101 | 0.368 |
| 30 | 0.616 | -0.761 | 99 | 0.442 | -0.290 | 168 | -0.410 | 0.310 |
| 31 | 0.436 | 1.619 | 100 | 1.594 | 1.333 | 169 | -0.435 | 0.059 |
| 32 | 0.824 | 1.648 | 101 | 0.738 | -0.507 | 170 | -0.656 | 0.509 |
| 33 | -1.009 | 0.475 | 102 | 0.357 | -0.600 | 171 | -0.901 | -0.330 |
| 34 | -0.700 | -0.714 | 103 | 1.870 | 0.645 | 172 | -0.962 | -0.096 |
| 35 | -0.192 | 0.326 | 104 | 0.492 | -0.735 | 173 | -0.537 | 0.527 |
| 36 | 0.636 | 1.166 | 105 | 0.970 | -0.345 | 174 | -1.125 | 0.048 |
| 37 | -0.763 | -1.259 | 106 | 0.678 | 1.639 | 175 | -0.935 | -0.481 |
| 38 | -0.579 | -1.183 | 107 | 3.177 | 1.004 | 176 | -0.901 | -0.424 |
| 39 | -1.607 | -0.509 | 108 | 1.090 | 0.810 | 177 | 0.367 | -0.074 |
| 40 | -1.124 | -0.479 | 109 | 1.377 | -1.088 | 178 | -0.054 | 0.270 |
| 41 | -0.720 | -0.703 | 110 | 1.285 | -1.024 | 179 | -0.490 | -0.620 |
| 42 | -0.174 | 0.002 | 111 | -1.904 | 1.496 | 180 | -2.111 | 0.190 |
| 43 | 0.548 | -0.147 | 112 | -1.915 | 1.382 | 181 | -0.223 | -0.203 |
| 44 | -0.637 | -0.188 | 113 | -2.358 | 1.034 | 182 | -1.188 | -0.183 |
| 45 | 1.038 | 0.415 | 114 | -4.413 | -0.092 | 183 | 0.838 | 1.887 |
| 46 | 2.921 | 0.842 | 115 | -0.781 | -0.383 | 184 | 0.301 | -2.962 |
| 47 | 0.373 | -0.801 | 116 | -0.527 | -0.271 | 185 | -0.790 | -1.421 |
| 48 | -0.100 | -0.811 | 117 | -1.500 | 0.244 | 186 | 0.361 | -0.513 |
| 49 | 1.396 | -0.753 | 118 | -0.625 | -0.358 | 187 | 1.883 | -1.291 |
| 50 | 0.930 | -1.363 | 119 | -0.470 | 1.313 | 188 | 3.870 | 0.358 |
| 51 | 0.445 | 1.042 | 120 | -0.283 | 2.179 | 190 | 0.836 | -1.176 |
| 52 | 0.759 | 0.838 | 121 | -0.029 | 1.560 | 191 | 3.682 | -1.692 |
| 53 | 0.530 | 1.452 | 122 | -0.595 | 2.210 | 192 | -0.017 | -0.302 |
| 54 | 1.651 | -0.164 | 123 | 0.593 | 1.199 | 193 | 0.368 | 0.708 |
| 55 | 0.545 | -1.332 | 124 | -0.730 | 0.594 | 194 | 0.471 | -2.021 |
| 56 | 0.092 | -0.317 | 125 | -1.402 | 1.000 | 195 | 0.437 | -1.784 |
| 57 | 0.043 | -0.137 | 126 | 0.002 | 0.863 | 196 | 0.546 | -1.413 |
| 58 | 0.253 | 0.502 | 127 | 0.578 | 0.108 | 198 | 0.373 | -1.565 |
| 59 | -0.486 | -1.054 | 128 | -0.483 | -0.160 | 199 | 0.864 | -1.312 |
| 60 | -0.969 | 0.489 | 129 | -0.661 | 1.481 | 200 | 0.459 | -2.988 |
| 61 | 1.227 | -0.550 | 130 | 1.090 | 1.322 | 201 | 1.469 | -2.857 |
| 62 | -0.646 | -0.049 | 131 | -0.938 | 1.313 | 202 | 0.972 | -1.462 |
| 63 | -1.075 | -1.590 | 132 | -0.011 | 0.138 | 204 | -0.461 | -1.473 |
| 64 | 1.148 | 0.187 | 133 | -1.406 | 0.372 | 205 | 1.270 | -2.536 |
| 65 | 0.431 | -1.386 | 134 | -0.213 | 0.154 | 206 | 0.088 | -0.879 |
| 66 | -0.252 | -1.000 | 135 | -0.510 | 1.413 |  |  |  |
| 67 | -0.205 | -0.825 | 136 | -0.356 | 1.200 |  |  |  |
| 68 | -0.355 | -0.095 | 137 | -0.501 | 2.287 |  |  |  |
| 69 | -0.220 | -0.192 | 138 | 0.360 | 1.275 |  |  |  |
