# Supplementary material for: Avian Wing Proportions and Flight Styles: First Step towards Predicting the Flight Modes of Mesozoic Birds
Source: PLoS One. 2011 Dec 7;6(12):e28672. doi: 10.1371/journal.pone.0028672 (PMC3233598; doi:10.1371/journal.pone.0028672)
Supplement: Table S3 — Results of Discriminant Function Analysis (DFA). (DOC) [file pone.0028672.s003.doc]

Table S3. Results of discriminant function analysis(DFA)

| **Casewise Statistics** | | | | | | | | | | | | | | | | | | |
| --- | --- | --- | --- | --- | --- | --- | --- | --- | --- | --- | --- | --- | --- | --- | --- | --- | --- | --- |
|  | Case Number | | Actual Group | | Highest Group | | | | | | | | | | Discriminant Scores | | |  |
|  |  |  |  |  | Predicted Group | | | P(D>d \| G=g) | | | | P(G=g \| D=d) | | Squared Mahalanobis Distance to Centroid | Function 1 | Function 2 | Function 3 |  |
|  |  |  |  |  |  |  |  | p | | df | |  |  |  |  |  |  |  |
| Original | 1 | | 1 | | 1 | | | .868 | | 3 | | .444 | | .720 | .366 | .306 | .413 |  |
|  | 2 | | 1 | | 1 | | | .442 | | 3 | | .962 | | 2.689 | 1.882 | -1.400 | -.558 |  |
|  | 3 | | 1 | | 4^**^ | | | .007 | | 3 | | .661 | | 12.087 | -.554 | -.734 | -3.443 |  |
|  | 4 | | 1 | | 1 | | | .304 | | 3 | | .976 | | 3.636 | 2.180 | -1.535 | -.468 |  |
|  | 5 | | 1 | | 4^**^ | | | .856 | | 3 | | .646 | | .771 | -1.508 | .524 | -.601 |  |
|  | 6 | | 1 | | 1 | | | .153 | | 3 | | .643 | | 5.265 | 2.154 | .658 | -1.614 |  |
|  | 7 | | 1 | | 4^**^ | | | .799 | | 3 | | .471 | | 1.011 | -.428 | -.754 | .178 |  |
|  | 8 | | 1 | | 1 | | | .184 | | 3 | | .982 | | 4.837 | 2.006 | -2.122 | .231 |  |
|  | 9 | | 1 | | 1 | | | .123 | | 3 | | .759 | | 5.782 | 2.464 | .410 | -1.615 |  |
|  | 10 | | 1 | | 1 | | | .175 | | 3 | | .954 | | 4.953 | 1.584 | -1.646 | -1.577 |  |
|  | 11 | | 1 | | 4^**^ | | | .845 | | 3 | | .410 | | .821 | -.340 | .652 | -.105 |  |
|  | 12 | | 1 | | 1 | | | .179 | | 3 | | .386 | | 4.910 | -.292 | -1.637 | 1.352 |  |
|  | 13 | | 1 | | 1 | | | .352 | | 3 | | .972 | | 3.266 | 1.918 | -1.709 | -.166 |  |
|  | 14 | | 1 | | 4^**^ | | | .686 | | 3 | | .479 | | 1.482 | -.797 | -1.163 | .043 |  |
|  | 15 | | 1 | | 1 | | | .181 | | 3 | | .881 | | 4.884 | .898 | -1.804 | -1.589 |  |
|  | 16 | | 1 | | 1 | | | .413 | | 3 | | .445 | | 2.863 | .145 | .015 | -1.513 |  |
|  | 17 | | 1 | | 1 | | | .667 | | 3 | | .592 | | 1.567 | .340 | -.385 | 1.155 |  |
|  | 18 | | 1 | | 1 | | | .250 | | 3 | | .471 | | 4.104 | -.091 | -1.008 | 1.659 |  |
|  | 19 | | 1 | | 1 | | | .932 | | 3 | | .860 | | .441 | 1.383 | -.592 | .193 |  |
|  | 20 | | 1 | | 4^**^ | | | .116 | | 3 | | .748 | | 5.911 | -1.169 | -.144 | -2.500 |  |
|  | 21 | | 1 | | 2^**^ | | | .802 | | 3 | | .888 | | .996 | -.064 | 2.257 | .349 |  |
|  | 22 | | 1 | | 1 | | | .291 | | 3 | | .969 | | 3.741 | 2.588 | -1.073 | .113 |  |
|  | 23 | | 1 | | 1 | | | .494 | | 3 | | .406 | | 2.397 | -.230 | -.913 | .943 |  |
|  | 24 | | 1 | | 2^**^ | | | .081 | | 3 | | .503 | | 6.721 | 2.371 | 1.103 | -1.570 |  |
|  | 25 | | 1 | | 1 | | | .148 | | 3 | | .958 | | 5.345 | 1.441 | -2.166 | -1.178 |  |
|  | 26 | | 1 | | 1 | | | .442 | | 3 | | .934 | | 2.688 | 1.306 | -1.605 | .843 |  |
|  | 27 | | 1 | | 4^**^ | | | .718 | | 3 | | .462 | | 1.349 | -1.101 | -1.121 | .018 |  |
|  | 28 | | 1 | | 1 | | | .464 | | 3 | | .941 | | 2.561 | 1.458 | -1.478 | .858 |  |
|  | 29 | | 1 | | 4^**^ | | | .537 | | 3 | | .449 | | 2.175 | -.748 | -1.417 | .038 |  |
|  | 30 | | 1 | | 1 | | | .148 | | 3 | | .980 | | 5.346 | 2.927 | -1.245 | -.049 |  |
|  | 31 | | 1 | | 4^**^ | | | .360 | | 3 | | .511 | | 3.216 | -.335 | .329 | -1.719 |  |
|  | 32 | | 1 | | 1 | | | .195 | | 3 | | .521 | | 4.706 | .375 | .005 | -2.095 |  |
|  | 33 | | 1 | | 4^**^ | | | .944 | | 3 | | .507 | | .384 | -1.557 | -.192 | .072 |  |
|  | 34 | | 1 | | 1 | | | .124 | | 3 | | .978 | | 5.753 | 1.722 | -2.494 | .218 |  |
|  | 35 | | 1 | | 1 | | | .293 | | 3 | | .934 | | 3.726 | 1.233 | -1.911 | -.939 |  |
|  | 36 | | 1 | | 1 | | | .448 | | 3 | | .351 | | 2.653 | .083 | .391 | -1.277 |  |
|  | 37 | | 1 | | 1 | | | .295 | | 3 | | .481 | | 3.705 | .131 | -.206 | 1.800 |  |
|  | 38 | | 1 | | 1 | | | .349 | | 3 | | .457 | | 3.292 | .135 | -.065 | 1.671 |  |
|  | 39 | | 1 | | 3^**^ | | | .687 | | 3 | | .634 | | 1.481 | -1.611 | -.412 | 1.413 |  |
|  | 40 | | 1 | | 4^**^ | | | .619 | | 3 | | .439 | | 1.783 | -.435 | -.675 | .902 |  |
|  | 41 | | 1 | | 1 | | | .709 | | 3 | | .613 | | 1.387 | .258 | -.731 | .934 |  |
|  | 42 | | 1 | | 1 | | | .856 | | 3 | | .412 | | .772 | .013 | -.030 | .222 |  |
|  | 43 | | 1 | | 1 | | | .322 | | 3 | | .971 | | 3.494 | 2.333 | -1.246 | -.532 |  |
|  | 44 | | 1 | | 1 | | | .200 | | 3 | | .943 | | 4.644 | 1.196 | -2.363 | -.393 |  |
|  | 45 | | 1 | | 2^**^ | | | .974 | | 3 | | .704 | | .224 | .573 | 1.325 | -.349 |  |
|  | 46 | | 1 | | 2^**^ | | | .280 | | 3 | | .921 | | 3.831 | 1.514 | 2.375 | -1.206 |  |
|  | 47 | | 1 | | 1 | | | .723 | | 3 | | .882 | | 1.325 | 1.857 | -.451 | .492 |  |
|  | 48 | | 1 | | 1 | | | .657 | | 3 | | .929 | | 1.611 | 1.571 | -1.109 | .614 |  |
|  | 49 | | 1 | | 2^**^ | | | .266 | | 3 | | .970 | | 3.957 | .456 | 2.880 | 1.424 |  |
|  | 50 | | 1 | | 1 | | | .031 | | 3 | | .988 | | 8.850 | 3.539 | -1.355 | .589 |  |
|  | 51 | | 1 | | 4^**^ | | | .663 | | 3 | | .427 | | 1.586 | -.414 | .853 | -.834 |  |
|  | 52 | | 1 | | 1 | | | .636 | | 3 | | .405 | | 1.704 | .452 | .542 | -.936 |  |
|  | 53 | | 1 | | 1 | | | .044 | | 3 | | .944 | | 8.082 | 1.510 | -1.571 | -2.431 |  |
|  | 54 | | 1 | | 2^**^ | | | .291 | | 3 | | .548 | | 3.740 | 2.305 | 1.127 | -.057 |  |
|  | 55 | | 1 | | 1 | | | .322 | | 3 | | .903 | | 3.491 | 2.348 | -.412 | 1.085 |  |
|  | 56 | | 1 | | 1 | | | .391 | | 3 | | .969 | | 3.002 | 1.896 | -1.617 | -.250 |  |
|  | 57 | | 1 | | 1 | | | .960 | | 3 | | .838 | | .298 | 1.053 | -.768 | -.076 |  |
|  | 58 | | 1 | | 1 | | | .822 | | 3 | | .399 | | .914 | .130 | .231 | -.402 |  |
|  | 59 | | 1 | | 1 | | | .567 | | 3 | | .587 | | 2.027 | .412 | -.236 | 1.368 |  |
|  | 60 | | 1 | | 4^**^ | | | .982 | | 3 | | .554 | | .174 | -1.197 | -.318 | .031 |  |
|  | 61 | | 1 | | 1 | | | .480 | | 3 | | .862 | | 2.475 | 2.387 | -.098 | -.094 |  |
|  | 62 | | 1 | | 4^**^ | | | .896 | | 3 | | .525 | | .604 | -.722 | .024 | .645 |  |
|  | 63 | | 1 | | 1 | | | .167 | | 3 | | .519 | | 5.061 | .118 | -.508 | 2.130 |  |
|  | 64 | | 1 | | 2^**^ | | | .962 | | 3 | | .765 | | .290 | .863 | 1.493 | -.177 |  |
|  | 65 | | 1 | | 1 | | | .234 | | 3 | | .946 | | 4.265 | 2.477 | -.774 | 1.148 |  |
|  | 66 | | 1 | | 1 | | | .378 | | 3 | | .967 | | 3.088 | 1.827 | -1.616 | .555 |  |
|  | 67 | | 1 | | 1 | | | .731 | | 3 | | .594 | | 1.294 | .536 | -.102 | 1.093 |  |
|  | 68 | | 1 | | 4^**^ | | | .844 | | 3 | | .434 | | .821 | -.308 | .114 | .499 |  |
|  | 69 | | 1 | | 1 | | | .754 | | 3 | | .368 | | 1.194 | -.018 | .157 | .545 |  |
|  | 70 | | 1 | | 1 | | | .607 | | 3 | | .618 | | 1.838 | .562 | -.178 | 1.333 |  |
|  | 71 | | 1 | | 2^**^ | | | .677 | | 3 | | .898 | | 1.523 | .902 | 2.143 | -.777 |  |
|  | 72 | | 1 | | 2^**^ | | | .516 | | 3 | | .615 | | 2.281 | .772 | 1.122 | -1.353 |  |
|  | 73 | | 1 | | 4^**^ | | | .734 | | 3 | | .436 | | 1.278 | -.318 | -.672 | .483 |  |
|  | 74 | | 1 | | 1 | | | .970 | | 3 | | .801 | | .243 | 1.319 | -.283 | -.033 |  |
|  | 75 | | 1 | | 2^**^ | | | .848 | | 3 | | .833 | | .807 | .562 | 1.815 | -.706 |  |
|  | 76 | | 1 | | 1 | | | .043 | | 3 | | .842 | | 8.163 | 3.462 | .258 | .976 |  |
|  | 77 | | 1 | | 1 | | | .210 | | 3 | | .951 | | 4.531 | 1.398 | -1.874 | 1.296 |  |
|  | 78 | | 1 | | 1 | | | .330 | | 3 | | .901 | | 3.431 | 1.377 | -.970 | -1.621 |  |
|  | 79 | | 1 | | 1 | | | .686 | | 3 | | .892 | | 1.483 | 1.059 | -1.397 | -.414 |  |
|  | 80 | | 1 | | 1 | | | .077 | | 3 | | .870 | | 6.850 | 3.396 | .156 | -.247 |  |
|  | 81 | | 1 | | 2^**^ | | | .942 | | 3 | | .544 | | .394 | .455 | .860 | .371 |  |
|  | 82 | | 1 | | 2^**^ | | | .714 | | 3 | | .845 | | 1.366 | 1.463 | 1.835 | .307 |  |
|  | 83 | | 1 | | 2^**^ | | | .998 | | 3 | | .734 | | .035 | .389 | 1.432 | -.103 |  |
|  | 84 | | 1 | | 1 | | | .815 | | 3 | | .381 | | .942 | .013 | .141 | -.312 |  |
|  | 85 | | 1 | | 2^**^ | | | .529 | | 3 | | .952 | | 2.215 | 1.104 | 2.555 | .723 |  |
|  | 86 | | 1 | | 1 | | | .265 | | 3 | | .821 | | 3.967 | .551 | -2.223 | .334 |  |
|  | 87 | | 1 | | 1 | | | .864 | | 3 | | .439 | | .738 | .017 | -.181 | .273 |  |
|  | 88 | | 1 | | 1 | | | .483 | | 3 | | .851 | | 2.459 | 1.304 | -.617 | 1.463 |  |
|  | 89 | | 1 | | 2^**^ | | | .358 | | 3 | | .830 | | 3.225 | 1.428 | 1.705 | 1.522 |  |
|  | 90 | | 1 | | 1 | | | .111 | | 3 | | .689 | | 6.022 | 2.497 | .496 | 1.632 |  |
|  | 91 | | 2 | | 2 | | | .730 | | 3 | | .428 | | 1.295 | -.257 | .773 | .758 |  |
|  | 92 | | 2 | | 2 | | | .607 | | 3 | | .831 | | 1.837 | .742 | 1.814 | -1.167 |  |
|  | 93 | | 2 | | 2 | | | .705 | | 3 | | .597 | | 1.400 | .748 | 1.055 | -.991 |  |
|  | 94 | | 2 | | 1^**^ | | | .842 | | 3 | | .384 | | .830 | -.001 | .099 | -.003 |  |
|  | 95 | | 2 | | 2 | | | .871 | | 3 | | .478 | | .711 | .307 | .708 | .534 |  |
|  | 96 | | 2 | | 2 | | | .837 | | 3 | | .699 | | .853 | 1.168 | 1.318 | -.418 |  |
|  | 97 | | 2 | | 4^**^ | | | .807 | | 3 | | .386 | | .975 | -.129 | .375 | -.395 |  |
|  | 98 | | 2 | | 4^**^ | | | .639 | | 3 | | .535 | | 1.693 | -1.288 | .992 | .748 |  |
|  | 99 | | 2 | | 2 | | | .894 | | 3 | | .710 | | .612 | .204 | 1.337 | .835 |  |
|  | 100 | | 2 | | 2 | | | .433 | | 3 | | .801 | | 2.743 | .433 | 1.763 | -1.537 |  |
|  | 101 | | 2 | | 2 | | | .858 | | 3 | | .753 | | .766 | .496 | 1.409 | .951 |  |
|  | 102 | | 2 | | 2 | | | .713 | | 3 | | .710 | | 1.370 | .054 | 1.366 | 1.199 |  |
|  | 103 | | 2 | | 2 | | | .686 | | 3 | | .852 | | 1.484 | 1.110 | 1.883 | -.787 |  |
|  | 104 | | 2 | | 2 | | | .714 | | 3 | | .715 | | 1.365 | .596 | 1.259 | 1.222 |  |
|  | 105 | | 2 | | 2 | | | .721 | | 3 | | .895 | | 1.336 | .131 | 2.165 | .918 |  |
|  | 106 | | 2 | | 4^**^ | | | .345 | | 3 | | .451 | | 3.320 | -.351 | .764 | -1.617 |  |
|  | 107 | | 2 | | 2 | | | .068 | | 3 | | .985 | | 7.121 | 1.729 | 3.424 | -1.060 |  |
|  | 108 | | 2 | | 2 | | | .906 | | 3 | | .769 | | .556 | -.010 | 1.717 | -.465 |  |
|  | 109 | | 2 | | 2 | | | .431 | | 3 | | .906 | | 2.756 | 1.071 | 2.077 | 1.446 |  |
|  | 110 | | 2 | | 2 | | | .422 | | 3 | | .887 | | 2.807 | 1.186 | 1.962 | 1.453 |  |
|  | 111 | | 3 | | 3 | | | .464 | | 3 | | .860 | | 2.561 | -3.439 | -.214 | -.520 |  |
|  | 112 | | 3 | | 3 | | | .582 | | 3 | | .889 | | 1.953 | -3.314 | -.767 | -.448 |  |
|  | 113 | | 3 | | 3 | | | .507 | | 3 | | .908 | | 2.331 | -3.615 | -.199 | .099 |  |
|  | 114 | | 3 | | 3 | | | .008 | | 3 | | .997 | | 11.757 | -4.840 | -2.687 | 1.141 |  |
|  | 115 | | 3 | | 4^**^ | | | .709 | | 3 | | .443 | | 1.386 | -.368 | -.406 | .811 |  |
|  | 116 | | 3 | | 4^**^ | | | .795 | | 3 | | .460 | | 1.024 | -.445 | .146 | .758 |  |
|  | 117 | | 3 | | 4^**^ | | | .659 | | 3 | | .445 | | 1.603 | -1.006 | -1.195 | .233 |  |
|  | 118 | | 3 | | 4^**^ | | | .752 | | 3 | | .460 | | 1.202 | -.449 | .045 | .868 |  |
|  | 119 | | 4 | | 4 | | | .517 | | 3 | | .576 | | 2.276 | -2.160 | .882 | -.532 |  |
|  | 120 | | 4 | | 4 | | | .227 | | 3 | | .735 | | 4.340 | -1.360 | -.033 | -2.131 |  |
|  | 121 | | 4 | | 4 | | | .536 | | 3 | | .667 | | 2.181 | -.853 | .071 | -1.549 |  |
|  | 122 | | 4 | | 4 | | | .143 | | 3 | | .687 | | 5.431 | -1.492 | -.582 | -2.274 |  |
|  | 123 | | 4 | | 4 | | | .535 | | 3 | | .426 | | 2.186 | -.390 | .872 | -1.133 |  |
|  | 124 | | 4 | | 4 | | | .984 | | 3 | | .563 | | .157 | -1.371 | -.100 | -.043 |  |
|  | 125 | | 4 | | 3^**^ | | | .627 | | 3 | | .543 | | 1.745 | -1.622 | -1.185 | -.706 |  |
|  | 126 | | 4 | | 4 | | | .887 | | 3 | | .586 | | .642 | -.931 | .714 | -.495 |  |
|  | 127 | | 4 | | 2^**^ | | | .959 | | 3 | | .697 | | .303 | -.107 | 1.462 | .299 |  |
|  | 128 | | 4 | | 4 | | | .803 | | 3 | | .487 | | .994 | -.631 | .402 | .769 |  |
|  | 129 | | 4 | | 4 | | | .600 | | 3 | | .547 | | 1.870 | -2.150 | .607 | -.545 |  |
|  | 130 | | 4 | | 2^**^ | | | .605 | | 3 | | .646 | | 1.847 | .138 | 1.321 | -1.250 |  |
|  | 131 | | 4 | | 4 | | | .742 | | 3 | | .520 | | 1.246 | -1.748 | -.412 | -.780 |  |
|  | 132 | | 4 | | 4 | | | .817 | | 3 | | .418 | | .935 | -.456 | .724 | .319 |  |
|  | 133 | | 4 | | 3^**^ | | | .821 | | 3 | | .637 | | .920 | -2.237 | .246 | .679 |  |
|  | 134 | | 4 | | 4 | | | .915 | | 3 | | .471 | | .518 | -.469 | .383 | .256 |  |
|  | 135 | | 4 | | 4 | | | .684 | | 3 | | .637 | | 1.494 | -1.722 | .230 | -1.046 |  |
|  | 136 | | 4 | | 4 | | | .848 | | 3 | | .661 | | .807 | -1.159 | -.002 | -.964 |  |
|  | 137 | | 4 | | 4 | | | .221 | | 3 | | .681 | | 4.406 | -1.573 | -.382 | -2.057 |  |
|  | 138 | | 4 | | 4 | | | .574 | | 3 | | .470 | | 1.991 | -.330 | .505 | -1.228 |  |
|  | 139 | | 4 | | 4 | | | .783 | | 3 | | .423 | | 1.076 | -.216 | -.575 | .226 |  |
|  | 140 | | 4 | | 4 | | | .793 | | 3 | | .511 | | 1.036 | -.361 | .022 | -.873 |  |
|  | 141 | | 4 | | 3^**^ | | | .907 | | 3 | | .717 | | .553 | -1.846 | -1.295 | .180 |  |
|  | 142 | | 4 | | 4 | | | .853 | | 3 | | .534 | | .787 | -.712 | -.796 | -.218 |  |
|  | 143 | | 4 | | 4 | | | .829 | | 3 | | .576 | | .884 | -1.760 | .585 | -.082 |  |
|  | 144 | | 4 | | 4 | | | .942 | | 3 | | .646 | | .394 | -1.186 | .122 | -.673 |  |
|  | 145 | | 4 | | 4 | | | .954 | | 3 | | .541 | | .333 | -.722 | .535 | -.132 |  |
|  | 146 | | 4 | | 4 | | | .910 | | 3 | | .633 | | .539 | -1.199 | -.178 | -.755 |  |
|  | 147 | | 4 | | 4 | | | .887 | | 3 | | .539 | | .642 | -1.413 | .277 | .560 |  |
|  | 148 | | 4 | | 4 | | | .995 | | 3 | | .573 | | .068 | -.914 | -.109 | .122 |  |
|  | 149 | | 4 | | 4 | | | .350 | | 3 | | .466 | | 3.282 | -2.076 | 1.080 | .932 |  |
|  | 150 | | 4 | | 4 | | | .836 | | 3 | | .488 | | .854 | -.528 | -.397 | .589 |  |
|  | 151 | | 4 | | 3^**^ | | | .926 | | 3 | | .566 | | .468 | -2.040 | -.067 | .058 |  |
|  | 152 | | 4 | | 3^**^ | | | .409 | | 3 | | .540 | | 2.886 | -1.587 | -1.441 | -1.018 |  |
|  | 153 | | 4 | | 4 | | | .827 | | 3 | | .504 | | .893 | -.840 | -.435 | .726 |  |
|  | 154 | | 4 | | 4 | | | .867 | | 3 | | .563 | | .724 | -.695 | -.645 | -.497 |  |
|  | 155 | | 4 | | 4 | | | .656 | | 3 | | .455 | | 1.617 | -.621 | -1.013 | .538 |  |
|  | 156 | | 4 | | 4 | | | .956 | | 3 | | .558 | | .321 | -1.024 | -.533 | -.122 |  |
|  | 157 | | 4 | | 4 | | | .863 | | 3 | | .536 | | .745 | -.951 | .118 | .777 |  |
|  | 158 | | 4 | | 4 | | | .924 | | 3 | | .505 | | .474 | -.541 | -.021 | .431 |  |
|  | 159 | | 4 | | 4 | | | .796 | | 3 | | .483 | | 1.020 | -.537 | -.010 | .816 |  |
|  | 160 | | 4 | | 4 | | | .865 | | 3 | | .611 | | .735 | -.857 | .479 | -.798 |  |
|  | 161 | | 4 | | 4 | | | .990 | | 3 | | .562 | | .118 | -1.177 | -.191 | .113 |  |
|  | 162 | | 4 | | 3^**^ | | | .869 | | 3 | | .478 | | .716 | -1.355 | -.750 | .224 |  |
|  | 163 | | 4 | | 4 | | | .426 | | 3 | | .491 | | 2.787 | -1.485 | .758 | 1.342 |  |
|  | 164 | | 4 | | 4 | | | .933 | | 3 | | .613 | | .434 | -.807 | .171 | -.695 |  |
|  | 165 | | 4 | | 4 | | | .552 | | 3 | | .505 | | 2.097 | -1.271 | 1.316 | .531 |  |
|  | 166 | | 4 | | 4 | | | .754 | | 3 | | .539 | | 1.195 | -1.122 | .611 | .838 |  |
|  | 167 | | 4 | | 4 | | | .738 | | 3 | | .498 | | 1.261 | -.933 | -1.089 | -.130 |  |
|  | 168 | | 4 | | 4 | | | .973 | | 3 | | .548 | | .225 | -.749 | .277 | .238 |  |
|  | 169 | | 4 | | 4 | | | .943 | | 3 | | .518 | | .385 | -.614 | .168 | .386 |  |
|  | 170 | | 4 | | 4 | | | .944 | | 3 | | .554 | | .383 | -.710 | -.508 | -.171 |  |
|  | 171 | | 4 | | 4 | | | .727 | | 3 | | .473 | | 1.311 | -.530 | -.345 | .893 |  |
|  | 172 | | 4 | | 4 | | | .894 | | 3 | | .518 | | .610 | -1.043 | -.294 | .628 |  |
|  | 173 | | 4 | | 4 | | | .976 | | 3 | | .594 | | .207 | -1.219 | .364 | .139 |  |
|  | 174 | | 4 | | 4 | | | .849 | | 3 | | .504 | | .801 | -.839 | -.734 | .355 |  |
|  | 175 | | 4 | | 4 | | | .321 | | 3 | | .473 | | 3.497 | -1.553 | .840 | 1.512 |  |
|  | 176 | | 4 | | 4 | | | .647 | | 3 | | .452 | | 1.656 | -.554 | -.836 | .758 |  |
|  | 177 | | 4 | | 2^**^ | | | .929 | | 3 | | .529 | | .451 | .168 | .879 | .418 |  |
|  | 178 | | 4 | | 4 | | | .880 | | 3 | | .424 | | .670 | -.323 | .460 | .088 |  |
|  | 179 | | 4 | | 4 | | | .299 | | 3 | | .450 | | 3.672 | -1.026 | .996 | 1.575 |  |
|  | 180 | | 4 | | 3^**^ | | | .421 | | 3 | | .651 | | 2.814 | -1.316 | -2.046 | -.003 |  |
|  | 181 | | 4 | | 1^**^ | | | .820 | | 3 | | .416 | | .921 | .036 | -.003 | .475 |  |
|  | 182 | | 4 | | 4 | | | .750 | | 3 | | .485 | | 1.212 | -1.275 | -.061 | .981 |  |
|  | 183 | | 4 | | 4 | | | .144 | | 3 | | .372 | | 5.407 | -.025 | .630 | -2.105 |  |
| **. Misclassified case | | | | | | | | | | | | | | | | | | |
| Classification Resultsa | | | | | | | | | | | | | | | | | | |
|  |  | VAR00001 | | Predicted Group Membership | | | | | | | Total | |  | | | | | |
|  |  |  |  | 1.00 | | 2.00 | 3.00 | | 4.00 | |  |  |  | | | | | |
| Original | Count | 1.00 | | 58 | | 15 | 1 | | 16 | | 90 | |  | | | | | |
|  |  | 2.00 | | 1 | | 16 | 0 | | 3 | | 20 | |  | | | | | |
|  |  | 3.00 | | 0 | | 0 | 4 | | 4 | | 8 | |  | | | | | |
|  |  | 4.00 | | 1 | | 3 | 7 | | 54 | | 65 | |  | | | | | |
|  | % | 1.00 | | 64.4 | | 16.7 | 1.1 | | 17.8 | | 100.0 | |  | | | | | |
|  |  | 2.00 | | 5.0 | | 80.0 | .0 | | 15.0 | | 100.0 | |  | | | | | |
|  |  | 3.00 | | .0 | | .0 | 50.0 | | 50.0 | | 100.0 | |  | | | | | |
|  |  | 4.00 | | 1.5 | | 4.6 | 10.8 | | 83.1 | | 100.0 | |  | | | | | |
| a. 72.1% of original grouped cases correctly classified. | | | | | | | | | | | | |  | | | | | |

| **Structure Matrix** | | | |
| --- | --- | --- | --- |
|  | Function | | |
|  | 1 | 2 | 3 |
| HU | -.887^*^ | .245 | .391 |
| PRI | .754^*^ | -.533 | .385 |
| UL | -.155 | .913^*^ | .377 |
| HAND^a^ | .064 | -.260 | -.963^*^ |
| Pooled within-groups correlations between discriminating variables and standardized canonical discriminant functions. | | | |
| *. Largest absolute correlation between each variable and any discriminant function.a, this variable was not used in the analysis. | | | |

| **Eigenvalues** | | | | |
| --- | --- | --- | --- | --- |
| Function | Eigenvalue | % of Variance | Cumulative % | Canonical Correlation |
| 1 | .938^a^ | 76.3 | 76.3 | .696 |
| 2 | .282^a^ | 22.9 | 99.3 | .469 |
| 3 | .009^a^ | .7 | 100.0 | .095 |
| a. First 3 canonical discriminant functions were used in the analysis. | | | | |

Stepwise Statistics

| **Variables Entered/Removed^a,b,c,d^** | | | | | | | | | | | | | |
| --- | --- | --- | --- | --- | --- | --- | --- | --- | --- | --- | --- | --- | --- |
| Step | Entered | Wilks' Lambda | | | | | | | | | | | |
|  |  | Statistic | df1 | df2 | df3 | Exact F | | | | Approximate F | | | |
|  |  |  |  |  |  | Statistic | df1 | df2 | Sig. | Statistic | df1 | df2 | Sig. |
| 1 | HU | .569 | 1 | 3 | 179.000 | 45.197 | 3 | 179.000 | .000 |  |  |  |  |
| 2 | UL | .431 | 2 | 3 | 179.000 | 31.039 | 6 | 356.000 | .000 |  |  |  |  |
| 3 | PRI | .399 | 3 | 3 | 179.000 |  |  |  |  | 21.985 | 9 | 430.922 | .000 |
| At each step, the variable that minimizes the overall Wilks' Lambda is entered. | | | | | | | | | | | | | |
| a. Maximum number of steps is 8. | | | | | | | | | | | | | |
| b. Minimum partial F to enter is 3.84. | | | | | | | | | | | | | |
| c. Maximum partial F to remove is 2.71. | | | | | | | | | | | | | |
| d. F level, tolerance, or VIN insufficient for further computation. | | | | | | | | | | | | | |
