# Supplementary material for: Avian Wing Proportions and Flight Styles: First Step towards Predicting the Flight Modes of Mesozoic Birds
Source: PLoS One. 2011 Dec 7;6(12):e28672. doi: 10.1371/journal.pone.0028672 (PMC3233598; doi:10.1371/journal.pone.0028672)
Supplement: Table S4 — Casewise statistics of fossil birds in Discriminant Function Analysis (DFA). (DOC) [file pone.0028672.s004.doc]

Table S4. Casewise statistics of fossil birds in discriminant function analysis (DFA).

| **Casewise Statistics of fossil birds** | | | | | | | | | | | |
| --- | --- | --- | --- | --- | --- | --- | --- | --- | --- | --- | --- |
|  | | Actual Group | Highest Group | | | | | Discriminant Scores | | |  |
|  |  |  | Predicted Group | P(D>d \| G=g) | | P(G=g \| D=d) | Squared Mahalanobis Distance to Centroid | Function 1 | Function 2 | Function 3 |  |
|  |  |  |  | p | df |  |  |  |  |  |  |
| Original | *Archaeopteryx* | Ar. | Ar. | 1.000 | 3 | .828 | .000 | -2.950 | -.245 | 2.240 |  |
|  | *Confuciusornis* | Co. | Co. | 1.000 | 3 | .818 | .000 | -.872 | -1.559 | 1.582 |  |
|  | *Eoenantiornis buhleri* | En. | CF | .823 | 3 | .298 | .910 | -.813 | .263 | .221 |  |
|  | *Alethoalaornis agitornis* | En. | En. | .709 | 3 | .555 | 1.385 | -2.577 | 1.909 | .408 |  |
|  | *Concornis lacustrus* | En. | CF | .000 | 3 | .668 | 32.527 | -4.483 | -2.584 | -3.622 |  |
|  | *Dapingfangornis sentisorhinus* | En. | Or. | .739 | 3 | .481 | 1.258 | -1.248 | 2.018 | .801 |  |
|  | *Eoalulavis hoyasi* | En. | En. | .008 | 3 | .741 | 11.787 | -3.663 | 3.728 | -.560 |  |
|  | *Longipteryx chaoyangensis* | En. | FS | .616 | 3 | .391 | 1.795 | .171 | 1.439 | .538 |  |
|  | *Longirostrornis hani* | En. | CF | .810 | 3 | .451 | .964 | .127 | -.297 | -.853 |  |
|  | *Protopteryx fengningensis* | En. | Or. | .845 | 3 | .400 | .820 | -2.419 | .714 | 1.435 |  |
|  | *Shanweiniao cooperorum* | En. | Or. | .984 | 3 | .470 | .157 | -1.772 | .693 | 1.302 |  |
|  | *Vescornis hebeiensis* | En. | CF | .876 | 3 | .370 | .689 | -1.917 | .165 | .830 |  |
|  | *Paraprotopteryx gracilisi* | En. | Or. | .956 | 3 | .543 | .319 | -1.234 | 1.409 | 1.197 |  |
|  | *Cathayornis sp.* | En. | En. | .958 | 3 | .430 | .313 | -1.761 | 1.338 | .575 |  |
|  | *Cuspirostrisornis houi* | En. | En. | .986 | 3 | .818 | .148 | -3.219 | -.043 | 2.054 |  |
|  | *Sinornis santensis* | En. | En. | .335 | 3 | .380 | 3.393 | -3.485 | 1.449 | 1.363 |  |
|  | *Hongshanornis longicresta* | Or. | En. | .970 | 3 | .497 | .245 | -2.300 | .466 | .460 |  |
|  | *Yixianornis grabaui* | Or. | Or. | .576 | 3 | .641 | 1.981 | -.534 | 1.585 | 1.795 |  |
|  | *Jianchangornis microdonta* | Or. | Or. | .521 | 3 | .484 | 2.786 | -2.820 | 1.823 | 1.521 |  |
|  | *Archaeorhynchus spathula* | Or. | CF | .727 | 3 | .267 | 1.508 | -.845 | .163 | .687 |  |

Ar.*Archaeopteryx*; Co. *Confuciusornis*; En. Enantiornithines; Or, Ornithurae; CF, ‘continuous flapping’; FS, ‘flapping and soaring’; FG, ‘flapping and gliding’; PT, ‘passerine-type flight’.
