# Supplementary material for: Correction: Avian Wing Proportions and Flight Styles: First Step towards Predicting the Flight Modes of Mesozoic Birds
Source: PLoS One. 2013 Oct 24;8(10):10.1371/annotation/6c8c755f-58fc-49e3-b499-8d082318eef6. doi: 10.1371/annotation/6c8c755f-58fc-49e3-b499-8d082318eef6 (PMC3815030; doi:10.1371/annotation/6c8c755f-58fc-49e3-b499-8d082318eef6)
Supplement: Supplementary file 1 [file pone.6c8c755f-58fc-49e3-b499-8d082318eef6.s001.doc]

**Wing proportions and flight style in birds: inferring the flight mode of Mesozoic fossil birds**

Xia Wang^1^, Alistair McGowan^2^ and Gareth J. Dyke^1,*^

**SUPPORTING MATERIAL**

Table S1 Measurements for living birds and fossil birds used in analyses.

| latin name   | flight-type | *hu* (mm) | *ul* (mm) | *mn* (mm) | *f_prim_(*mm) | *ta*(mm) | *M* (g) |  | | --- | --- | --- | --- | --- | --- | --- | --- | | *Acanthis cannabina* | PT | 17.03 | 20.99 | 13.93 | 43 | 51.95 | 15.30 | | *Accipiter gentilis* | FS | 94.21 | 105.05 | 78.86 | 273 | 278.12 | 1024.50 | | *Accipiter nisus* | FS | 51.77 | 62.29 | 49.74 | 146 | 163.8 | 237.50 | | *Acrocephalus schoenobaenus* | PT | 12.69 | 15.17 | 12.38 | 63 | 40.24 | 11.20 | | *Acrocephalus scirpaceus* | PT | 11.93 | 16.84 | 14.97 | 57 | 43.74 | 12.30 | | *Aegithalos caudatus* | PT | 11 | 13.85 | 11 | 45 | 35.85 | 8.20 | | *Aix galericulata* | CF | 70.1 | 58 | 45.6 | 191 | 173.7 | 570.00 | | *Alauda arvensis* | PT | 25.16 | 30.63 | 24.01 | 85 | 79.8 | 39.95 | | *Alca torda* | CF | 76.91 | 59 | 48.87 | 131 | 184.78 | 67.75 | | *Alcedo atthis* | PT | 25.16 | 29.45 | 14.74 | 62 | 69.35 | 719.00 | | *Alectoris rufa* | CF | 53.43 | 49.8 | 41.15 | 121 | 144.38 | 483.00 | | *Alle alle* | CF | 43.56 | 35.17 | 28.54 | 92 | 107.27 | 163.00 | | *Anas acuta* | CF | 90.6 | 78.78 | 95.52 | 234 | 264.9 | 1010.50 | | *Anas clypeata* | CF | 75.04 | 64.53 | 51.63 | 208 | 191.2 | 613.00 | | *Anas crecca* | CF | 59.63 | 47.9 | 50.91 | 156 | 158.44 | 341.00 | | *Anas penelope* | CF | 86.22 | 72.51 | 72.87 | 197 | 231.6 | 771.50 | | *Anas platyrhynchos* | CF | 89.75 | 74.25 | 63.84 | 204 | 227.84 | 1082.00 | | *Anser anser* | CF | 179.74 | 171.11 | 181.19 | 410 | 532.04 | 3308.50 | | *Anthus pratensis* | PT | 19.52 | 23.85 | 16.65 | 78 | 60.02 | 18.40 | | *Apus apus* | FG | 11.83 | 18.11 | 27.93 | 146 | 57.87 | 22.75 | | *Aquila chrysaetos* | FS | 184.56 | 214.78 | 95.47 | 405 | 494.81 | 4197.00 | | *Ardea cinerea* | CF | 170.73 | 200.77 | 120.78 | 284 | 492.28 | 1443.00 | | *Ardea purpurea* | CF | 138.42 | 159.15 | 76.59 | 258 | 374.16 | 935.00 | | *Arenaria interpres* | CF | 39.91 | 43.12 | 44.74 | 136 | 127.77 | 115.00 | | *Asio flammeus* | CF | 83.57 | 100.73 | 59 | 246 | 243.3 | 346.50 | | *Asio otus* | CF | 80.4 | 92.14 | 48.88 | 250 | 221.42 | 262.00 | | *Athene noctua* | CF | 54.08 | 69.8 | 41.96 | 123 | 165.84 | 164.00 | | *Aythya ferina* | CF | 86.46 | 72.37 | 71.44 | 153 | 230.27 | 823.00 | | *Aythya fuligula* | CF | 74.55 | 67.33 | 57.05 | 148 | 198.93 | 694.00 | | *Bombycilla garrulus* | PT | 22.2 | 27.4 | 15.5 | 101 | 65.1 | 56.40 | | *Botaurus stellaris* | FS | 134.29 | 143.18 | 81.91 | 258 | 359.38 | 916.50 | | *Branta bernicla* | CF | 123.57 | 113.08 | 122.47 | 410 | 359.12 | 1300.00 | | *Branta canadensis* | CF | 180.77 | 161.33 | 99.5 | 438 | 441.6 | 2943.07 | | *Branta leucopsis* | CF | 132.53 | 120.55 | 99.72 | 358 | 352.8 | 1687.00 | | *Bubo bubo* | CF | 157.79 | 183.77 | 112.13 | 358 | 453.69 | 2686.00 | | *Bubulcus ibis* | FS | 94 | 106.88 | 50.15 | 190 | 251.03 | 338.00 | | *Bucephala clangula* | CF | 69.8 | 59.08 | 55.7 | 205 | 184.58 | 900.00 | | *Burhinus oedicnemus* | CF | 80.26 | 90.34 | 74.88 | 230 | 245.48 | 459.00 | | *Buteo buteo* | FS | 106.88 | 127.15 | 94.35 | 253 | 328.38 | 875.00 | | *Buteo lagopus* | FS | 112.92 | 131.31 | 97.73 | 247 | 341.96 | 956.00 | | *Calidris alpina* | CF | 28.07 | 29.36 | 36.39 | 105 | 93.82 | 47.75 | | *Calidris canutus* | CF | 43.02 | 47.36 | 52.04 | 123 | 142.42 | 137.00 | | *Caprimulgus europaeus* | CF | 37.74 | 48.51 | 53.31 | 167 | 139.56 | 67.00 | | *Carduelis carduelis* | PT | 16.85 | 22.98 | 21.19 | 63 | 61.02 | 44.65 | | *Carduelis chloris* | PT | 19.03 | 23.37 | 15.28 | 74 | 57.68 | 27.80 | | *Carduelis spinus* | PT | 12.98 | 15.94 | 15.71 | 68 | 44.63 | 14.50 | | *Cepphus grylle* | CF | 60.18 | 50.46 | 34.2 | 150 | 144.84 | 405.00 | | *Certhia familiaris* | PT | 12.8 | 15.8 | 8.37 | 53 | 36.97 | 9.00 | | *Charadrius hiaticula* | CF | 32.66 | 36.07 | 31.84 | 115 | 100.57 | 64.00 | | *Ciconia ciconia* | FS | 199.33 | 229.92 | 169.04 | 350 | 598.29 | 3473.00 | | *Ciconia nigra* | FS | 196 | 217 | 107.33 | 359 | 520.33 | 3000.00 | | *Cinclus cinclus* | PT | 22 | 24.9 | 19.2 | 82 | 66.1 | 59.80 | | *Circus aeruginosus* | FS | 104.11 | 126.34 | 100.43 | 253 | 330.88 | 627.50 | | *Circus cyaneus* | FS | 92.34 | 104.42 | 79.37 | 230 | 276.13 | 435.50 | | *Circus pygargus* | FS | 86.58 | 116.88 | 92.76 | 303 | 296.22 | 315.50 | | *Coccothraustes coccothraustes* | PT | 23.64 | 26.77 | 23.64 | 98 | 74.05 | 54.00 | | *Columba livia* | CF | 44.62 | 51.34 | 47 | 193 | 142.96 | 354.50 | | *Columba oenas* | CF | 46.8 | 51.03 | 48.4 | 193 | 146.23 | 291.00 | | *Columba palumbus* | CF | 54.9 | 59.02 | 26.21 | 208 | 140.13 | 490.00 | | *Coracias garrulus* | PT | 45.4 | 57.44 | 54.93 | 179 | 157.77 | 146.00 | | *Corvus corone* | CF | 66.26 | 79.5 | 69.63 | 228 | 215.39 | 570.00 | | *Corvus frugilegus* | CF | 66.98 | 81.93 | 67.68 | 270 | 216.59 | 488.00 | | *Corvus monedula* | CF | 47.36 | 60.46 | 49.92 | 204 | 157.74 | 275.00 | | *Coturnix coturnix* | CF | 35.76 | 29.94 | 23.63 | 80 | 89.33 | 96.50 | | *Crex crex* | CF | 48.88 | 43.51 | 46.73 | 126 | 139.12 | 155.50 | | *Cuculus canorus* | PT | 39.74 | 44.04 | 45.81 | 201 | 129.59 | 113.00 | | *Cygnus olor* | CF | 279.68 | 254.68 | 231.93 | 423 | 766.29 | 10735.00 | | *Delichon urbica* | FG | 13.98 | 21.3 | 16.57 | 95 | 51.85 | 14.50 | | *Dryocopus martius* | PT | 54 | 60 | 50 | 156 | 164 | 321.00 | | *Egretta alba* | CF | 152.4 | 177.53 | 91.23 | 320 | 421.16 | 1100.00 | | *Emberiza cirlus* | PT | 17.7 | 19.65 | 19.37 | 67 | 56.72 | 23.10 | | *Emberiza citrinella* | PT | 20.52 | 24.04 | 20.25 | 68 | 64.81 | 26.50 | | *Emberiza schoeniclus* | PT | 17.96 | 20.59 | 18.77 | 62 | 57.32 | 18.30 | | *Erithacus rubecula* | PT | 16.39 | 19.52 | 15.01 | 51 | 50.92 | 18.20 | | *Falco columbarius* | FG | 47.58 | 53.82 | 50.95 | 158 | 152.35 | 190.50 | | *Falco peregrinus* | FG | 87.26 | 102.32 | 92.39 | 280 | 281.97 | 781.50 | | *Falco subbuteo* | FG | 55.32 | 63.03 | 60.5 | 236 | 178.85 | 240.00 | | *Falco tinnunculus* | FG | 53.85 | 63.41 | 55.37 | 170 | 172.63 | 201.50 | | *Ficedula hypoleuca* | PT | 14.85 | 21.82 | 18.1 | 62 | 54.77 | 11.60 | | *Fratercula arctica* | CF | 63.26 | 50.77 | 42.78 | 114 | 156.81 | 381.00 | | *Fringilla coelebs* | PT | 18.16 | 22.43 | 20.01 | 65 | 60.6 | 21.40 | | *Fringilla montifringilla* | PT | 18.87 | 22.86 | 20.83 | 83 | 62.56 | 24.00 | | *Fulica atra* | CF | 77.33 | 66.98 | 62.42 | 135 | 206.73 | 892.50 | | *Gallinago gallinago* | CF | 37.5 | 39.77 | 39.38 | 105 | 116.65 | 122.00 | | *Gallinula chloropus* | CF | 50.68 | 41.64 | 36.85 | 116 | 129.17 | 302.50 | | *Garrulus glandarius* | PT | 41.68 | 49.06 | 36.06 | 125 | 126.8 | 161.00 | | *Gavia immer* | CF | 191.68 | 153.05 | 99.74 | 250 | 444.47 | 4134.00 | | *Gavia stellata* | CF | 139.63 | 112.89 | 91.33 | 174 | 343.85 | 1551.00 | | *Grus grus* | CF | 227.16 | 247.46 | 192.5 | 384 | 667.12 | 5500.00 | | *Gypaetus barbatus* | FS | 225.81 | 266.36 | 135.88 | 670 | 628.05 | 5680.00 | | *Gyps fulvus* | FS | 237.85 | 292.02 | 161.55 | 576 | 691.42 | 7436.00 | | *Haematopus ostralegus* | CF | 73.47 | 77.75 | 72.91 | 190 | 224.13 | 526.00 | | *Haliaeetus albicilla* | FS | 220.24 | 248.99 | 186.2 | 381 | 655.43 | 4793.00 | | *Hirundo rustica* | FG | 14.98 | 23.41 | 20.56 | 105 | 58.95 | 16.00 | | *Jynx torquilla* | PT | 23.41 | 27.25 | 15.51 | 71 | 66.17 | 33.50 | | *Lagopus lagopus* | CF | 67.13 | 59.94 | 57.13 | 154 | 184.2 | 558.50 | | *Lagopus mutus* | CF | 60.83 | 54.1 | 38.03 | 181 | 152.96 | 422.00 | | *Lanius collurio* | PT | 20.18 | 23.85 | 19.6 | 80 | 63.63 | 29.90 | | *Lanius excubitor* | PT | 26.86 | 33.02 | 20.77 | 92 | 80.65 | 65.60 | | *Lanius senator* | PT | 21.65 | 29.66 | 21.65 | 73 | 72.96 | 29.10 | | *Larus argentatus* | CF | 128.77 | 144.26 | 103.82 | 305 | 376.85 | 1135.00 | | *Larus canus* | CF | 83.05 | 91.64 | 84.48 | 282 | 259.17 | 403.50 | | *Larus marinus* | CF | 153.27 | 170.55 | 120.16 | 442 | 443.98 | 1658.50 | | *Larus ridibundus* | CF | 76.81 | 88.81 | 73.77 | 237 | 239.39 | 284.00 | | *Limosa lapponica* | CF | 60.59 | 64.53 | 65.17 | 163 | 190.29 | 342.50 | | *Limosa limosa* | CF | 62.46 | 67.19 | 38.21 | 180 | 167.86 | 291.00 | | *Loxia curvirostra* | PT | 20.5 | 25.24 | 20.77 | 81 | 66.51 | 36.50 | | *Luscinia megarhynchos* | PT | 17 | 22.93 | 18.81 | 81 | 58.74 | 19.63 | | *Luscinia svecica* | PT | 16.02 | 20.87 | 17.72 | 63 | 54.61 | 16.63 | | *Lymnocryptes minimus* | CF | 31.75 | 33.98 | 40.79 | 105 | 106.52 | 50.20 | | *Mergus serrator* | CF | 89.03 | 72.07 | 55.3 | 176 | 216.4 | 1021.50 | | *Milvus migrans* | FS | 117.24 | 133.85 | 90.43 | 340 | 341.52 | 278.00 | | *Milvus milvus* | FS | 123.58 | 141.42 | 112.82 | 268 | 377.82 | 1080.00 | | *Monticola solitarius* | PT | 25.84 | 35.77 | 33.79 | 97 | 95.4 | 53.75 | | *Motacilla alba* | PT | 19.21 | 23.9 | 16.28 | 74 | 59.39 | 22.70 | | *Motacilla cinerea* | PT | 18.07 | 23.77 | 15.08 | 78 | 56.92 | 17.60 | | *Muscicapa striata* | PT | 15.4 | 21.7 | 14.55 | 70 | 51.65 | 14.60 | | *Neophron percnopterus* | PT | 143.45 | 161.65 | 75.95 | 401 | 381.05 | 2120.00 | | *Numenius arquata* | CF | 96.01 | 104 | 61.04 | 240 | 261.05 | 805.50 | | *Nyctea scandiaca* | CF | 156 | 169 | 84 | 340 | 409 | 2042.50 | | *Nycticorax nycticorax* | CF | 107.31 | 119.48 | 70.37 | 216 | 297.16 | 883.00 | | *Oriolus oriolus* | PT | 31.95 | 40.84 | 31.45 | 119 | 104.24 | 79.00 | | *Otis tarda* | CF | 196.46 | 215.42 | 177.16 | 582 | 589.04 | 8100.00 | | *Otus scops* | CF | 67.07 | 86.41 | 57.75 | 124 | 211.23 | 92.00 | | *Pandion haliaetus* | FS | 146.4 | 184.99 | 124.7 | 319 | 456.09 | 1600.00 | | *Parus ater* | PT | 12.7 | 15.1 | 8.7 | 46 | 36.5 | 9.10 | | *Parus caeruleus* | PT | 13.95 | 17.01 | 14.45 | 45 | 45.41 | 13.30 | | *Parus major* | PT | 16.42 | 20 | 14.86 | 49 | 51.28 | 19.00 | | *Parus montanus* | PT | 13.6 | 16.3 | 9 | 40 | 38.9 | 10.20 | | *Passer domesticus* | PT | 19.09 | 22.45 | 20.29 | 63 | 61.83 | 27.70 | | *Passer montanus* | PT | 16.79 | 18.92 | 15.71 | 64 | 51.42 | 22.00 | | *Pavo cristatus* | CF | 128.07 | 107.97 | 61.6 | 326 | 297.64 | 4187.50 | | *Pelecanus onocrotalus* | FS | 325 | 371 | 139.2 | 430 | 835.2 | 9600.00 | | *Perdix perdix* | CF | 51.92 | 45.78 | 48.37 | 127 | 146.07 | 389.50 | | *Phalacrocorax aristotelis* | CF | 122.3 | 133.5 | 56.5 | 193 | 312.3 | 1769.00 | | *Phalacrocorax carbo* | CF | 161.11 | 174.07 | 105.57 | 269 | 440.75 | 2109.50 | | *Phoenicurus phoenicurus* | PT | 15.99 | 20.34 | 14.81 | 75 | 51.14 | 14.60 | | *Pica pica* | CF | 43.09 | 51.31 | 35.4 | 127 | 129.8 | 177.50 | | *Picoides major* | PT | 32.02 | 36.79 | 29.58 | 118 | 98.39 | 81.60 | | *Picoides minor* | PT | 21.2 | 24.09 | 15.65 | 68 | 60.94 | 19.80 | | *Picus viridis* | PT | 41.55 | 48.08 | 35.03 | 105 | 124.66 | 176.00 | | *Plectrophenax nivalis* | PT | 20.2 | 23.65 | 18.85 | 103 | 62.7 | 42.20 | | *Plegadis falcinellus* | FS | 93.3 | 101.3 | 54.5 | 205 | 249.1 | 532.50 | | *Pluvialis apricaria* | CF | 48.66 | 54.41 | 54.56 | 169 | 157.63 | 214.00 | | *Pluvialis squatarola* | CF | 52.5 | 54.78 | 54.6 | 156 | 161.88 | 220.00 | | *Podiceps auritus* | CF | 77.65 | 67.93 | 33.85 | 124 | 179.43 | 453.00 | | *Podiceps cristatus* | CF | 108.05 | 102.2 | 62.28 | 162 | 272.53 | 673.50 | | *Podiceps grisegena* | CF | 107.14 | 98.82 | 45.22 | 161 | 251.18 | 1023.00 | | *Podiceps nigricollis* | CF | 69.68 | 63.88 | 31 | 116 | 164.56 | 292.00 | | *Prunella modularis* | PT | 16.44 | 17.99 | 15.55 | 54 | 49.98 | 20.25 | | *Pyrrhula pyrrhula* | PT | 18.94 | 23.27 | 16.39 | 62 | 58.6 | 21.80 | | *Rallus aquaticus* | CF | 40.24 | 32.12 | 32.37 | 94 | 104.73 | 120.00 | | *Recurvirostra avosetta* | CF | 73.48 | 77.27 | 76.55 | 190 | 227.3 | 306.00 | | *Regulus regulus* | PT | 8.82 | 12.72 | 8.22 | 39 | 29.76 | 5.70 | | *Riparia riparia* | FG | 14.5 | 21.12 | 17.89 | 102 | 53.51 | 14.60 | | *Rissa tridactyla* | CF | 85.6 | 94.6 | 64.48 | 238 | 244.68 | 407.00 | | *Saxicola rubetra* | PT | 16.18 | 22.06 | 16.05 | 67 | 54.29 | 16.60 | | *Saxicola torquata* | PT | 15.75 | 19.88 | 12.84 | 53 | 48.47 | 13.25 | | *Scolopax rusticola* | CF | 52.9 | 57.42 | 69.91 | 156 | 180.23 | 309.50 | | *Sitta europaea* | PT | 18.53 | 22.32 | 18.08 | 68 | 58.93 | 22.00 | | *Somateria mollissima* | CF | 111.8 | 97.13 | 69.23 | 190 | 278.16 | 2066.50 | | *Stercorarius parasiticus* | CF | 94 | 97.8 | 49.65 | 258 | 241.45 | 464.50 | | *Streptopelia turtur* | CF | 36.92 | 41.14 | 37.78 | 149 | 115.84 | 132.00 | | *Strix aluco* | CF | 84.01 | 95.03 | 55.8 | 185 | 234.84 | 475.00 | | *Sturnus vulgaris* | PT | 27.77 | 33.73 | 30.97 | 107 | 92.47 | 82.30 | | *Sylvia atricapilla* | PT | 16.97 | 20.01 | 19 | 63 | 55.98 | 15.50 | | *Sylvia curruca* | PT | 13.1 | 15.8 | 8.8 | 60 | 37.7 | 10.10 | | *Tetrao tetrix* | CF | 82.86 | 78.86 | 63.89 | 208 | 225.61 | 1082.50 | | *Tetrao urogallus* | CF | 118.24 | 113.12 | 85.95 | 263 | 317.31 | 2950.00 | | *Thalasseus sandvicensis* | CF | 69 | 82.5 | 41.2 | 278 | 192.7 | 208.00 | | *Tichodroma muraria* | PT | 19.98 | 25.9 | 21.95 | 70 | 67.83 | 17.20 | | *Tringa totanus* | CF | 44.6 | 49.41 | 52.07 | 125 | 146.08 | 129.00 | | *Troglodytes aedon* | CF | 13.18 | 14.42 | 11.3 | 34 | 38.9 | 10.90 | | *Turdus iliacus* | PT | 26.51 | 31.88 | 21.25 | 93 | 79.64 | 61.20 | | *Turdus merula* | PT | 29.67 | 35.43 | 28.82 | 98 | 93.92 | 93.77 | | *Turdus philomelos* | PT | 26.83 | 30.95 | 29.72 | 92 | 87.5 | 67.75 | | *Turdus pilaris* | PT | 29.97 | 34.58 | 31.81 | 116 | 96.36 | 106.00 | | *Turdus torquatus* | PT | 30.73 | 40.82 | 37.61 | 100 | 109.16 | 109.00 | | *Turdus viscivorus* | PT | 31.24 | 35.02 | 35.02 | 114 | 101.28 | 115.00 | | *Tyto alba* | CF | 82.52 | 91.41 | 51.75 | 230 | 225.68 | 447.00 | | *Upupa epops* | CF | 33.44 | 44.06 | 29.06 | 132 | 106.56 | 61.40 | | *Uria aalge* | CF | 87.25 | 63.13 | 58.89 | 130 | 209.27 | 992.50 | | *Vanellus vanellus* | CF | 61.91 | 68.55 | 54.72 | 181 | 185.18 | 218.50 | |  |  |  |  |  |  |  |
| --- | --- | --- | --- | --- | --- | --- | --- | --- | --- | --- | --- | --- | --- | --- | --- | --- | --- | --- | --- | --- | --- | --- | --- | --- | --- | --- | --- | --- | --- | --- | --- | --- | --- | --- | --- | --- | --- | --- | --- | --- | --- | --- | --- | --- | --- | --- | --- | --- | --- | --- | --- | --- | --- | --- | --- | --- | --- | --- | --- | --- | --- | --- | --- | --- | --- | --- | --- | --- | --- | --- | --- | --- | --- | --- | --- | --- | --- | --- | --- | --- | --- | --- | --- | --- | --- | --- | --- | --- | --- | --- | --- | --- | --- | --- | --- | --- | --- | --- | --- | --- | --- | --- | --- | --- | --- | --- | --- | --- | --- | --- | --- | --- | --- | --- | --- | --- | --- | --- | --- | --- | --- | --- | --- | --- | --- | --- | --- | --- | --- | --- | --- | --- | --- | --- | --- | --- | --- | --- | --- | --- | --- | --- | --- | --- | --- | --- | --- | --- | --- | --- | --- | --- | --- | --- | --- | --- | --- | --- | --- | --- | --- | --- | --- | --- | --- | --- | --- | --- | --- | --- | --- | --- | --- | --- | --- | --- | --- | --- | --- | --- | --- | --- | --- | --- | --- | --- | --- | --- | --- | --- | --- | --- | --- | --- | --- | --- | --- | --- | --- | --- | --- | --- | --- | --- | --- | --- | --- | --- | --- | --- | --- | --- | --- | --- | --- | --- | --- | --- | --- | --- | --- | --- | --- | --- | --- | --- | --- | --- | --- | --- | --- | --- | --- | --- | --- | --- | --- | --- | --- | --- | --- | --- | --- | --- | --- | --- | --- | --- | --- | --- | --- | --- | --- | --- | --- | --- | --- | --- | --- | --- | --- | --- | --- | --- | --- | --- | --- | --- | --- | --- | --- | --- | --- | --- | --- | --- | --- | --- | --- | --- | --- | --- | --- | --- | --- | --- | --- | --- | --- | --- | --- | --- | --- | --- | --- | --- | --- | --- | --- | --- | --- | --- | --- | --- | --- | --- | --- | --- | --- | --- | --- | --- | --- | --- | --- | --- | --- | --- | --- | --- | --- | --- | --- | --- | --- | --- | --- | --- | --- | --- | --- | --- | --- | --- | --- | --- | --- | --- | --- | --- | --- | --- | --- | --- | --- | --- | --- | --- | --- | --- | --- | --- | --- | --- | --- | --- | --- | --- | --- | --- | --- | --- | --- | --- | --- | --- | --- | --- | --- | --- | --- | --- | --- | --- | --- | --- | --- | --- | --- | --- | --- | --- | --- | --- | --- | --- | --- | --- | --- | --- | --- | --- | --- | --- | --- | --- | --- | --- | --- | --- | --- | --- | --- | --- | --- | --- | --- | --- | --- | --- | --- | --- | --- | --- | --- | --- | --- | --- | --- | --- | --- | --- | --- | --- | --- | --- | --- | --- | --- | --- | --- | --- | --- | --- | --- | --- | --- | --- | --- | --- | --- | --- | --- | --- | --- | --- | --- | --- | --- | --- | --- | --- | --- | --- | --- | --- | --- | --- | --- | --- | --- | --- | --- | --- | --- | --- | --- | --- | --- | --- | --- | --- | --- | --- | --- | --- | --- | --- | --- | --- | --- | --- | --- | --- | --- | --- | --- | --- | --- | --- | --- | --- | --- | --- | --- | --- | --- | --- | --- | --- | --- | --- | --- | --- | --- | --- | --- | --- | --- | --- | --- | --- | --- | --- | --- | --- | --- | --- | --- | --- | --- | --- | --- | --- | --- | --- | --- | --- | --- | --- | --- | --- | --- | --- | --- | --- | --- | --- | --- | --- | --- | --- | --- | --- | --- | --- | --- | --- | --- | --- | --- | --- | --- | --- | --- | --- | --- | --- | --- | --- | --- | --- | --- | --- | --- | --- | --- | --- | --- | --- | --- | --- | --- | --- | --- | --- | --- | --- | --- | --- | --- | --- | --- | --- | --- | --- | --- | --- | --- | --- | --- | --- | --- | --- | --- | --- | --- | --- | --- | --- | --- | --- | --- | --- | --- | --- | --- | --- | --- | --- | --- | --- | --- | --- | --- | --- | --- | --- | --- | --- | --- | --- | --- | --- | --- | --- | --- | --- | --- | --- | --- | --- | --- | --- | --- | --- | --- | --- | --- | --- | --- | --- | --- | --- | --- | --- | --- | --- | --- | --- | --- | --- | --- | --- | --- | --- | --- | --- | --- | --- | --- | --- | --- | --- | --- | --- | --- | --- | --- | --- | --- | --- | --- | --- | --- | --- | --- | --- | --- | --- | --- | --- | --- | --- | --- | --- | --- | --- | --- | --- | --- | --- | --- | --- | --- | --- | --- | --- | --- | --- | --- | --- | --- | --- | --- | --- | --- | --- | --- | --- | --- | --- | --- | --- | --- | --- | --- | --- | --- | --- | --- | --- | --- | --- | --- | --- | --- | --- | --- | --- | --- | --- | --- | --- | --- | --- | --- | --- | --- | --- | --- | --- | --- | --- | --- | --- | --- | --- | --- | --- | --- | --- | --- | --- | --- | --- | --- | --- | --- | --- | --- | --- | --- | --- | --- | --- | --- | --- | --- | --- | --- | --- | --- | --- | --- | --- | --- | --- | --- | --- | --- | --- | --- | --- | --- | --- | --- | --- | --- | --- | --- | --- | --- | --- | --- | --- | --- | --- | --- | --- | --- | --- | --- | --- | --- | --- | --- | --- | --- | --- | --- | --- | --- | --- | --- | --- | --- | --- | --- | --- | --- | --- | --- | --- | --- | --- | --- | --- | --- | --- | --- | --- | --- | --- | --- | --- | --- | --- | --- | --- | --- | --- | --- | --- | --- | --- | --- | --- | --- | --- | --- | --- | --- | --- | --- | --- | --- | --- | --- | --- | --- | --- | --- | --- | --- | --- | --- | --- | --- | --- | --- | --- | --- | --- | --- | --- | --- | --- | --- | --- | --- | --- | --- | --- | --- | --- | --- | --- | --- | --- | --- | --- | --- | --- | --- | --- | --- | --- | --- | --- | --- | --- | --- | --- | --- | --- | --- | --- | --- | --- | --- | --- | --- | --- | --- | --- | --- | --- | --- | --- | --- | --- | --- | --- | --- | --- | --- | --- | --- | --- | --- | --- | --- | --- | --- | --- | --- | --- | --- | --- | --- | --- | --- | --- | --- | --- | --- | --- | --- | --- | --- | --- | --- | --- | --- | --- | --- | --- | --- | --- | --- | --- | --- | --- | --- | --- | --- | --- | --- | --- | --- | --- | --- | --- | --- | --- | --- | --- | --- | --- | --- | --- | --- | --- | --- | --- | --- | --- | --- | --- | --- | --- | --- | --- | --- | --- | --- | --- | --- | --- | --- | --- | --- | --- | --- | --- | --- | --- | --- | --- | --- | --- | --- | --- | --- | --- | --- | --- | --- | --- | --- | --- | --- | --- | --- | --- | --- | --- | --- | --- | --- | --- | --- | --- | --- | --- | --- | --- | --- | --- | --- | --- | --- | --- | --- | --- | --- | --- | --- | --- | --- | --- | --- | --- | --- | --- | --- | --- | --- | --- | --- | --- | --- | --- | --- | --- | --- | --- | --- | --- | --- | --- | --- | --- | --- | --- | --- | --- | --- | --- | --- | --- | --- | --- | --- | --- | --- | --- | --- | --- | --- | --- | --- | --- | --- | --- | --- | --- | --- | --- | --- | --- | --- | --- | --- | --- | --- | --- | --- | --- | --- | --- | --- | --- | --- | --- | --- | --- | --- | --- | --- | --- | --- | --- | --- | --- | --- | --- | --- | --- | --- | --- | --- | --- | --- | --- | --- | --- | --- | --- | --- | --- | --- | --- | --- | --- | --- | --- | --- | --- | --- | --- | --- | --- | --- | --- | --- | --- | --- | --- | --- | --- | --- | --- | --- | --- | --- | --- | --- | --- | --- | --- | --- | --- | --- | --- | --- | --- | --- | --- | --- | --- | --- | --- | --- | --- | --- | --- | --- | --- | --- | --- | --- | --- | --- | --- | --- | --- | --- | --- | --- | --- | --- | --- | --- | --- | --- | --- | --- | --- | --- | --- | --- | --- | --- | --- | --- | --- | --- | --- | --- | --- | --- | --- | --- | --- | --- | --- | --- | --- | --- | --- | --- | --- | --- | --- | --- | --- | --- | --- | --- | --- | --- | --- | --- | --- | --- | --- | --- | --- | --- | --- | --- | --- | --- | --- | --- | --- | --- | --- | --- | --- | --- | --- | --- | --- | --- | --- | --- | --- | --- | --- | --- | --- | --- | --- | --- | --- | --- | --- | --- | --- | --- | --- | --- | --- | --- | --- | --- | --- | --- | --- | --- | --- | --- | --- | --- | --- | --- | --- | --- | --- | --- | --- | --- | --- | --- | --- | --- | --- | --- | --- | --- | --- | --- | --- | --- | --- | --- | --- | --- | --- | --- | --- | --- | --- | --- | --- | --- | --- | --- | --- | --- | --- | --- | --- | --- | --- | --- | --- | --- | --- | --- | --- | --- | --- | --- | --- | --- | --- | --- | --- | --- | --- | --- | --- | --- | --- | --- | --- | --- | --- | --- | --- | --- | --- | --- | --- | --- | --- | --- | --- | --- | --- | --- | --- | --- | --- | --- | --- | --- | --- | --- | --- | --- | --- | --- | --- | --- | --- | --- | --- | --- | --- | --- | --- | --- | --- | --- | --- | --- | --- | --- | --- | --- | --- | --- | --- | --- | --- | --- | --- | --- | --- | --- | --- | --- | --- | --- | --- | --- | --- | --- | --- | --- | --- | --- | --- | --- | --- | --- | --- | --- | --- | --- | --- | --- | --- | --- | --- | --- | --- | --- | --- | --- | --- | --- | --- | --- | --- | --- | --- | --- | --- | --- | --- | --- | --- | --- | --- | --- | --- | --- | --- | --- | --- | --- | --- | --- | --- | --- | --- | --- | --- | --- | --- | --- | --- | --- |
|  |  |  |  |  |  |  |  |
|  |  |  |  |  |  |  |  |
|  |  |  |  |  |  |  |  |

| latin name | Specimen No./Source | Humerus (mm) | forearm (mm) | hand (mm) | *fprim(mm)* | *ta(mm)* |
| --- | --- | --- | --- | --- | --- | --- |
| *Archaeopteryx* | [17] | 61.93 | 54.91 | 71.48 | 97.30 | 188.32 |
| *Confuciusornis* | pers.obs. | 49.32 | 44.84 | 55.09 | 138.79 | 149.25 |
| *Eoenantiornis buhleri* | IVPP V11537/pers.obs | 29.43 | 31.17 | 24.86 | 66.91 | 85.46 |
| *Alethoalaornis agitornis* | LPM 00009/[1] | 24.00 | 26.00 | 19.60 | 33.30 | 69.6 |
| *Concornis lacustrus* | LH2814/[2] | 68.10 | 38.90 | 15.00 | 47.70 | 122 |
| *Dapingfangornis sentisorhinus* | LPM 00039[3] | 22.00 | 27.00 | 22.20 | 44.40 | 71.2 |
| *Eoalulavis hoyasi* | LH 13500/[4] | 30.00 | 35.10 | 19.00 | 27.30 | 84.1 |
| *Longipteryx chaoyangensis* | STM A8-3/pers.obs. | 28.50 | 36.77 | 29.52 | 82.84 | 94.79 |
| *Longirostravis hani* | IVPP V11309/pers.obs. | 23.28 | 24.81 | 16.02 | 62.96 | 64.11 |
| *Protopteryx fengningensis* | IVPP V11665/pers.obs. | 26.89 | 26.76 | 27.40 | 44.09 | 81.05 |
| *Shanweiniao cooperorum* | DNHM D1878/1/[5] | 22.43 | 23.46 | 23.36 | 43.20 | 69 .25 |
| *Vescornis hebeiensis* | NIGP 130722/[6] | 25.20 | 24.60 | 22.60 | 45.70 | 72.4 |
| *Paraprotopteryx gracilisi* | STM 1-7/pers.obs. | 18.39 | 21.45 | 20.00 | 39.38 | 59.84 |
| *Cathayornis sp.* | STM A11-58/pers.obs. | 21.12 | 23.25 | 18.87 | 37.58 | 63.24 |
| *Cuspirostrisornis houi* | STM A11-65/pers.obs. | 26.49 | 23.36 | 28.70 | 38.25 | 78.55 |
| *131.27* | STM A11-66/pers.obs. | 44.84 | 44.06 | 42.37 | 54.21 | 131.27 |
| *Hongshanornis longicresta* | IVPP V14533/pers.obs. | 25.96 | 25.08 | 20.80 | 40.74 | 71.84 |
| *Yixianornis grabaui* | IVPP V12631/pers.obs. | 38.84 | 49.06 | 52.06 | 104.05 | 139.96 |
| *Jianchangornis microdonta* | IVPP V16708/pers.obs. | 76.20 | 82.10 | 80.30 | 109.80 | 238.6 |
| *Archaeorhynchus spathula* | IVPP V14287/pers.obs. | 53.00 | 56.00 | 50.00 | 125.00 | 159 |

Abbreviations: *hu*, humerus; *ul*, ulna; *mn*, manus; *f*_prim_, average primary feather length; *ta*, average total arm length (humerus+ulna+hand); *M*, body mass.Four flight styles -CF, ‘continuous flapping’; FS, ‘flapping and soaring’; FG, ‘flapping and gliding’; PT, ‘passerine-type flight’; Institutional acronyms (China): DNHM, Dalian Natural History Museum (Dalian); GMV, NGMC, National Geological Museum of China (Beijing); IVPP, Institute of Vertebrate Paleontology and Paleoanthropology (Beijing); LH, refers to the collection from Las Hoyas; LPM, Liaoning Provincial Museum (Beipiao); NIGP, Nanjing Institute of Geology and Paleontology, Chinese Academy of Science (Nanjing); STM, Shandong Tianyu Museum of Nature (Pingyi).

**Additional references**

1. Li, L., Hu, D.-Y., Duan, Y., Gong, E.-P.& Hou, L.-H. 2007 Alethoalaornithidae Fam. Nov: a new family of enantiornithine bird from the lower Cretaceous of western Liaoning. *Acta Palaeontologica Sinica* **46**, 365-372.
2. Sanz, J, L,, Chiappe, L. M. & Buscalioni, A. 1995 The osteology of *Concornis lacustris* (Aves: Enantiornithes) from the Lower Cretaceous of Spain and a re-examination of its phylogenetic relationships. *Am. Mus. Novit.* **3133**, 1–23.
3. Li, L., Duan, Y., Hu, D.-Y., Wang, L., Cheng, S.-L. & Hou, L.-H. 2006 New Eoenantiornithid bird from the Early Cretaceous Jiufotang Formation of western Liaoning, China. *Acta Geologica Sinica* (English edition) **80**, 38–41.
4. Sanz, J. L., Chiappe, L. M., Pérez-Moreno B. P., Buscalioni, A. D. & Moratalla, J. 1996 A Lower Cretaceous bird from Spain: implications for the evolution of flight. *Nature* **382**, 442–445.
5. O'Connor, J. K., Wang, X.-R., Chiappe, L. M., Gao, C.-H., Meng, Q.-J., Cheng, X.-D., & Liu, J.-Y. 2009 Phylogenetic support for a specialized clade of Cretaceous enantiornithine birds with information from a new species. *Journal of Vertebrate Paleontology* **29**, 188–204.
6. Zhang, F.-C., Ericson, P.G.P. & Zhou, Z.-H. 2004 Description of a new enantiornithine bird from the Early Cretaceous of Hebei, northern China. *Canadian Journal of Earth Sciences* **41**, 1097-1107.
